# Virulence of the emerging pathogen, *Burkholderia pseudomallei*, depends upon the *O*-linked oligosaccharyltransferase, PglL

**DOI:** 10.2217/fmb-2019-0165

**Published:** 2020-03-01

**Authors:** Samuel J Willcocks, Carmen Denman, Felipe Cia, Elizabeth McCarthy, Jon Cuccui, Brendan W Wren

**Affiliations:** 1 The London School of Hygiene & Tropical Medicine, WC1E 7HT, London, UK

**Keywords:** *Burkholderia*, glycosylation, oligosaccharyltransferase, PglL, secretion system, virulence

## Abstract

**Aim:**

We sought to characterize the contribution of the *O*-OTase, PglL, to virulence in two *Burkholderia* spp. by comparing isogenic mutants in *Burkholderia pseudomallei* with the related species, *Burkholderia thailandensis*.

**Materials & methods:**

We utilized an array of *in vitro* assays in addition to *Galleria mellonella* and murine *in vivo* models to assess virulence of the mutant and wild-type strains in each *Burkholderia* species.

**Results:**

We found that *pglL* contributes to biofilm and twitching motility in both species. *PglL* uniquely affected morphology; cell invasion; intracellular motility; plaque formation and intergenus competition in *B. pseudomallei*. This mutant was attenuated in the murine model, and extended survival in a vaccine-challenge experiment.

**Conclusion:**

Our data support a broad role for *pglL* in bacterial fitness and virulence, particularly in *B. pseudomallei*.

The Burkholderiaceae family are comprised of an interesting group of mostly soil-dwelling saprophytes. They have evolved diverse characteristics: some are able to fix nitrogen and are considered symbionts, others cause rot in important food crops; some are bioremediators, and others are opportunistic pathogens of animals [1]. Melioidosis, the potentially fatal disease for humans caused by *Burkholderia pseudomallei*, is highly endemic in Southeast Asia and northern Australia, and it is now clear that the bacteria can be isolated from soil found in many subtropical countries around the world [2–5]. Climate change may yet introduce the organism to regions where it was previously absent [6]. Hence, melioidosis can be considered both a neglected and an emerging public health concern. *B. pseudomallei* is also considered a tier one select agent by the Centres for Disease Control and Prevention. Understanding why this species in particular is so virulent in humans is therefore a research priority. While we have uncovered many of the molecular aspects that facilitate pathogenesis [7], the potential contribution of post-translational protein glycosylation to virulence is comparatively under studied. Previously considered solely a feature of higher organisms, protein glycosylation is now understood to be common among prokaryotes [8], and can contribute to protein folding, stability, trafficking, function and immunogenicity [9].

Glycosylation systems are primarily defined by two aspects: first, the type of bond that connects the glycan to the amino-acid – a covalent link to either an amide nitrogen of an asparagine residue (*N*’-linkcd glycosylation), or to a hydroxyl oxygen usually in a serine/threonine residue (*O*-linked glycosylation). Secondly, the mechanism by which the glycan is coupled to the peptide. This occurs either iteratively, with monosaccharides sequentially attached to an acceptor protein, a process enabled by a cytosolic glycosyltransferase; or as *en bloc* transfer of polysaccharides from a lipid carrier, typically UndPP [10], enabled by a periplasmic OTase [11,12].

PglL is one of the few types of *O*-linked OTases so far discovered; it is homologous to PilO in *Pseudomonas aeruginosa*, but PilO is limited to the transfer of relatively short chain-length oligosaccharides [12]. PglL was first identified in a *Burkholderia* spp. in 2012 [13], and recently has been shown to be well conserved across the genus, typically modifying peptides with two trisaccharide glycans: HexNAc-HexNAc-262 and HexNAc-HexNAc-Hex [14]

Detection of novel *O*-OTases is hampered by the fact that they contain a Wzy-C domain in common with those found in WaaL ligases [15], often leading to mis-annotation. Thus, *O*-OTase activity must be demonstrated experimentally for definitive assignment. By expressing meningococcal PglL in *Escherichia coli*, it was demonstrated that this enzyme could glycosylate Type IV pilin, microfilamentous cell-surface structures that confer twitching motility [12]. Pilin glycosylation, which can also be mediated by PilO, has been shown to contribute to: biofilm formation [16]; host-cell attachment [17]; defense against bacteriophage [18]; and resistance to surfactant proteins [19]. Interestingly, *Acinetobacter* spp. strains have recently been discovered that express more than one type of pilin glycoprotein [20], and some possess two functional PglL proteins, one thought to be dedicated to pilin glycosylation and the other to multiple other glycoproteins [21]. It is interesting to note that although *pglL* itself is conserved, the number and identity of glycoproteins dependent on its activity differs between *Burkholderia* species. For example, 23 unique glycoproteins have been identified in *Burkholderia cenocepacia* dependent on *pglL* [11], with 29 in *Burkholderia gladioli;* eight in *Burkholderia thailandensis* and ten in *B. pseudomallei* [14]. In *B. cencocepacia* these glycoproteins contribute to diverse functions including cell division; stress response; membrane transport; and virulence. A specific *ΔpglL* mutant in *B. cenocepacia* has been shown to have an altered phenotype relating to aspects of virulence, metabolic activity and sensitivity to oxidative and osmotic stress [14].

The available evidence suggests that PglL might play an influential role in the microbiology and virulence of *B. pseudomallei*. We therefore constructed unmarked deletion mutants of *pglL* for comparison in the mainly avirulent species, *B. thailandensis* E264 *(bth-0650)* and in *B. pseudomallei* K9264 *(bpsl0783)*. Through characterization in a range of *in vitro* and *in vivo* assays, we sought to understand its potential contribution to fitness and virulence. We report that *pglL* is required for virulence in *Burkholderia* spp. in a species-restricted manner, including the discovery of novel phenotypes not previously attributed to the function of this gene.

## Materials & methods

### Cell lines & culture conditions

RAW 264.7 murine macrophage-like cells and A549 human epithelial cell lines were both sourced from ATCC (LGC Standards, Manchester, UK) and maintained in sterile RPMI1640 Glutamax supplemented with 10% bovine fetal calf serum (both Thermo Fisher Scientific, Gloucester, UK). Cell were cultured at 37°C in an atmosphere of 5% CO_2_.

### *sacB* mediated unmarked gene deletion mutagenesis

For *B. thailandensis ΔpglL* mutagenesis, 1 kb downstream and upstream homologous DNAfragments to *BTHI0650 (pglLBhai)* were amplified using Phusion High-Fidelity polymerase chain reaction (PCR) master mix with GC buffer with 3% dimethyl sulfoxide (DMSO) (Finnzyme, #F532) from purified genomic DNA (DNAEasy, Qiagen). These fragments were assembled in a splicing-by-overlapping-extension PCR (SOE-PCR) and purified using a gel extraction kit (Qiagen). For *B. pseudomallei (pg^lL^Ps)*, the upstream and downstream dsDNA fragment was synthesized by GenScript (NJ, USA).

The inserts were ligated into the pJET1.2 blunt cloning vector (Fermentas*#K1231*) and transformation was performed on chemically competent *E. coli* Top10 cells (Invitrogen #C4040-50). Clones were screened by PCR and sequencing. The deletion construct was subcloned into the *Xba*I-digested dephosphorylated pDM4 vector via the restriction sites and transformed into *pir+ E. coli* EC100D (Epicenter) for antibiotic selection of positive colonies in LB agar with chloramphenicol. The pDM4 construct was then subcloned into electrocompetent *E. coli* MFD cells for conjugation with *Burkholderia* spp.

Overnight cultures of the donor strain and the recipient strain were washed once with sterile phosphate-buffered saline (PBS) and mixed in a 1:3 volume ratio. 100 μl of the mixture was applied to a prewarmed LB agar plate containing diaminopimelic acid (DAP) and incubated for no longer than 16 h at 37°C. Cells were resuspended and serially diluted in PBS and cultured overnight at 37°C on selective LB agar containing chloramphenicol. *B*. *thailandensis* and *B. pseudomallei* colonies were screened for presence of the deletion fragment.

Overnight culture of the merodiploid strain was diluted in PBS and 100 μl was plated on salt-free LB agar containing 10–12% (w/v) sucrose. The plate was incubated at 24°C until colonies were well grown. Colonies were screened for chloramphenicol sensitivity; independent sensitive colonies were then screened first using colony PCR and then selected for whole-genome next generation sequencing (performed by Public Health England, Genomic Services and Development Unit, London, UK) to confirm deletion at the intended site (Supplementary Figure 4).

### Antibiotic & complement assays

For each condition, wild-type *B. pseudomallei* K96243; *B*. *thailandensis* E264; *B. pseudomallei ΔpglL* and *B. thailandensis ΔpglL* were cultured overnight from a single colony in 5 ml sterile Luria–Bertani (LB) broth at 37°C with shaking. All strains employed in this study exhibited comparable growth rates when cultured in the media used. Bacteria concentrations were standardized by adjusting to an OD_590_ of 1 and then further diluted 1:100 in fresh LB. A total of 10 μl of bacterial solutions were added in triplicate to flat bottom 96-well plates containing 100 μl of titrated polymixin B or human serum (both, Sigma-Aldrich, Gillingham, UK) at the concentrations described. For heat inactivation, human serum was incubated at 65°C for 1 h. After 24 h incubation at 37°C, colony-forming unit (CFU) assay was performed by serial dilution of each sample well in sterile phosphate-buffered saline (PBS; Sigma, UK) before plating on fresh LB-agar and incubation for a further 24 h at 37°C for colony enumeration.

### Biofilm & twitching motility assay

Biofilm formation was assessed using the 96-well plate and accompanying peg-lid of the MBEC assay device (Innovotech, Farnborough, UK), as described by Denman and Brown [22]. Cultures were incubated statically at 37^°^C (biofilm assay) or at room temperature (twitching assay). To assess twitching motility, bacteria were first streaked onto sterile LB agar and incubated overnight. A small portion of similar size from each strain was picked from the outer edge of the colonies with a sterile 10 μl pipette tip and homogenized. The tip was then used to perpendicularly stab the centre of triplicate fresh 1% LB agar plates, and moved up and down three times to ensure bacterial transfer. Plates were inverted and incubated at room temperature (RT) until a visible interstitial colony could be observed between the plastic of the Petri dish and the agar layer. The agar was then removed from the Petri dish, and the diameter of the colonies on the plastic measured for calculation of average circumference.

### Cell infection assay

Either A549 epithelial cells or RAW 264.7 cells were seeded at a concentration of 1 × 10^5^ per 100 μl in sterile flat bottom 96-well plates in RPMI, 10% fetal calf serum (FCS) overnight at 37°C, 5% CO_2_. These cells were then infected for 90 min with fresh *Burkholderia* strains cultured overnight at a multiplicity of infection (MOI) of five. At 90 min (T = 0), cells were washed three times with sterile PBS prior to cell lysis with 0.5% Triton X100 in water for 30 min followed by mechanical disruption by pipetting. For later time points, cells were instead incubated in a medium of RPMI 10% FCS with 100 μg ml^-1^ kanamycin to control extracellular replication, this dose has been shown by others to completely kill extracellular *B*. *pseudomallei* and *B*. *thailandensis* [23]. After lysis at each time point, as well as from the input infection solution, bacterial CFU were enumerated by serial dilution and culture on LB agar overnight at 37^°^C.

### Confocal microscopy

A549 epithelial cells were seeded at a concentration of 1 × 10^5^ cells per 100 μl RPMI 10% FCS into Lab-Tek II Chamber Slides (Thermo Fisher Scientific, Gloucester, UK) and incubated overnight at 37°C with 5% CO_2_. These were infected with different MOI of each *Burkholderia* strain as indicated. After 90 min incubation at 37^°^C, the monolayer was washed with sterile PBS and media replaced with fresh RPMI 10% FCS with 100 μg ml^-1^ kanamycin to control extracellular replication. Chamber slides were then incubated for a further 20 h at 37°C with 5% CO_2_. Media was gently removed and the cells washed twice with sterile PBS before fixation with freshly prepared 4% paraformaldehyde for 1 h. Cells were permeabilized using 0.1% Triton X100 in PBS for 15 min at RT and then washed twice with PBS. Fcγ receptor was blocked using 10% goat serum for 30 min before incubation with primary antibody (a kind gift from Prof Richard Titball, University of Exeter, UK) for 1 h prior to two PBS washes and incubation with AlcxaFluor-488 goat anti-mouse IgG (H+L) secondary antibody (Thermo Fisher Scientific) along with 20 units/well AlexaFluor-546 phalloidin (Thermo Fisher Scientific) for 1 h. To mount the cells, the media was drained and the chambers removed from the glass slide. Monolayers were allowed to dry briefly before addition of Vectashield anti-fade mounting medium with DAPI (Vector Laboratories, Peterborough, UK) and attachment of the coverslip. All images were captured using a Zeiss LSM 880 confocal microscope with Plan-Apochromat 63x/1.4 Oil DIC M27 using standard confocal mode driven by ZEN Black 2.3 software (Carl Zeiss). 2x3 tile scan z-stacks were captured and stitched together using a 10% overlap. DAPI was visualized using a 405 nm laser and collection of light at 410–495 nm; phalloidin was visualized using a 561 nm laser and collection of light at 574–712 nm and AlexaFluor-488 was visualized using a 488 nm laser with collection of light at 499—571 nm.

Plaque quantification was determined manually, with values representing the average number from duplicate wells of an eight-well chamber slide. We defined a plaque as a space greater than 100 μM in diameter absent of any A549 cells. For actin tail length measurement, we utilized Zeiss Zen 2.3 software tools.

### Competition assay

To prepare *E*. *coli*, chemically competent TOP10 strains (Thermo Fisher Scientific) were transformed with pcDNA 3.3 TOPO-LacZ and plated onto dry LB agar precoated with 50 μl X-gal at 40 μg ml^-1^. A blue colony was selected and cultured overnight in 5 ml LB broth containing 100 μg ml^-1^ ampicillin. All *Burkholderia* colonies were cultured in LB broth. Optical density of all strains were standardized to an OD590 of one, centrifuged and resuspended in 200 μl sterile LB broth. A total of 30 μl of *E*. *coli* were mixed with 30 μl *Burkholderia* spp. from each strain in triplicate. These mixtures were cultured for 5 h at 37°C either in static liquid LB broth, or spotted onto LB agar. After 5 h the bacteria growing on agar were scraped and resuspended in 1 ml sterile PBS. For all conditions, including the input cultures, serial dilution was performed and bacteria spotted onto X-gal LB agar for CFU enumeration and calculation of blue:white *(E. coli:Burkholderia* spp.) ratios.

### *Galleria mellonella* larval infection

Following growth of bacteria in LB, infection of larvae with 1 × 10^4^ CFU was performed as described by Wand *et al*. [22,24,25], and larval survival monitored over a 72 h period. Live versus dead was determined by melanisation, and response to touch with a pipette tip.

### Mouse infection & vaccination

For all murine studies, female BALB/c mice (Charles Rivers Laboratories International, Kent, UK) were used with the approval of the local Ethical Review Committee and in accordance with the Animals (Scientific Procedures) Act of 1986. For each infection, mice were first anesthetized intraperitoneally with a ketamine (100 mg kg^-1^; Ketalar, Pfizer Ltd, Surrey, UK) and xylazine (10 mg kg^-1^; Rompun, UK) solution diluted in sterile saline. Infection inocula were administered by pipetting a total of 50 μl into both nostrils.

For vaccination, mice were first inoculated intranasally with varying CFU of *B*. *pseudomallei* Δ*pglL* mutant or sterile PBS as described with six mice per group. After 4 weeks, all mice were challenged with 500 CFU wildtype *B*. *pseudomallei* K96243. Surviving mice at the end of the experiment were culled to enable post-mortem enumeration of bacteria in lungs and spleen by CFU assay. PCR was conducted on the resultant colonies using Advantage GC DNA polymerase (Takara Bio, Clontech, UK) with the manufacturer’s recommended protocolto confirm the proportion of wild-type versus mutant strain by amplification of the *pglL* gene (forward primer: *5’* AACGTGCACTACCATCTCGG 3’; reverse primer: 5’ TTTCGCGAGCAGATCGATGA 3’).

### Statistical analysis of data

All numerical data were collated and analyzed using GraphPad Prism 8.1.2. or Microsoft Excel 2016. Individual statistical tests are described in the figure legends. Unless otherwise stated, figures represent the means of triplicate technical repeats from three independent biological replicates.

## Results

### *PglL* genetics

We compared the arrangement of genes close to *pglL* in *B*. *thailandensis* E264 and *B*. *pseudomallei* K9264 (using ATCC 700388 and GenBank assembly accession GCF-000011545.1, respectively; Supplementary Figure 1 & Supplementary Table 1), and found them to be highly syntenic, with an identical arrangement of upstream and downstream genes. As with the majority of the *Burkholderia* strains with publicly available genomes, a pili/fimbrae related family protein is located directly upstream of the *pglL* coding sequence. We also conducted a phylogenetic analysis of the PglL amino acid sequences alongside other *Burkholderia* spp. (Supplementary Figure 2) and found several clusters. PglL from *B*. *thailandensis* was more closely related to orthologues from species that can cause infection in humans and horses, *B*. *pseudomallei* and *Burkholderia mallei*, respectively, than it was to members of the *Burkholderia* complex that mainly consist of plant pathogens and saprophytes. Finally, we examined amino acid conservation and found PglL to be 95% identical over 96% of the sequence, although some nonsynonymous mutations, insertions and deletions were identified (Supplementary Figure 3).

### PglL_Burk_ contributes to biofilm formation & twitching motility

**Figure 1:**
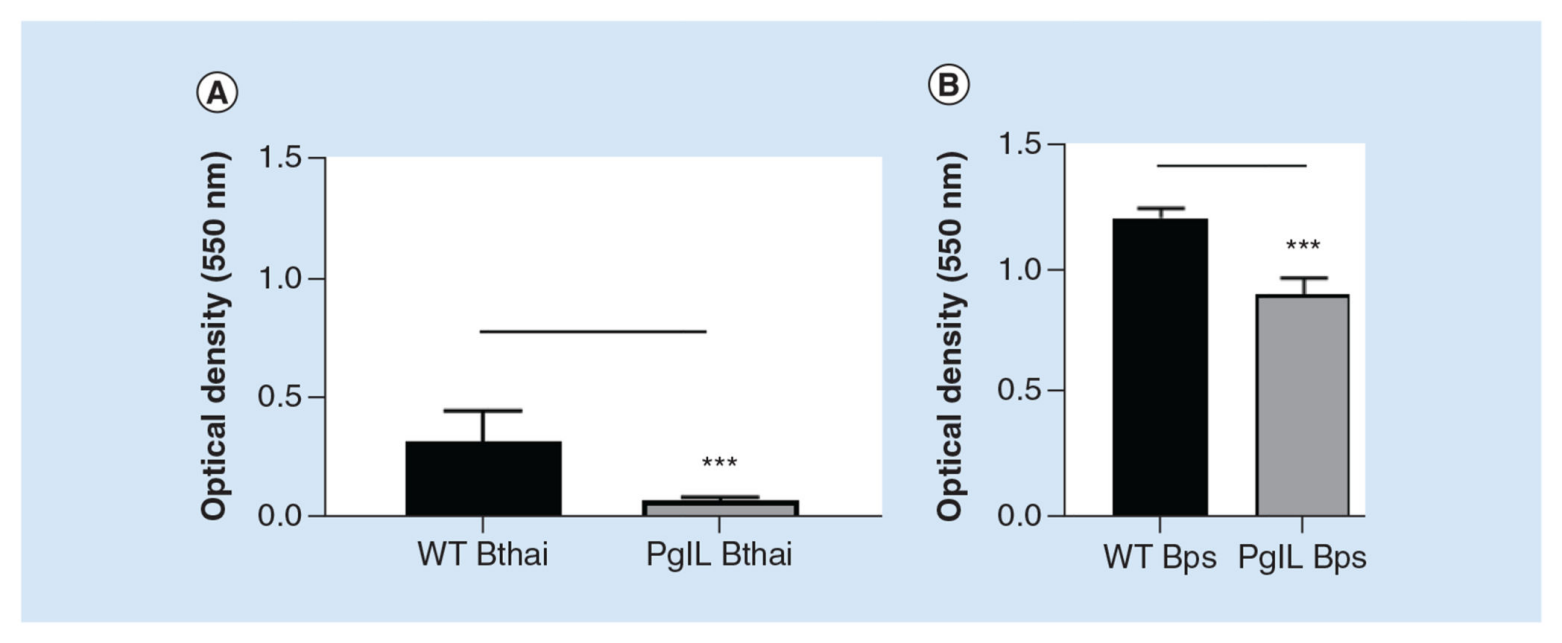
Biofilm formation in wild-type and Δ*pglL Burkholderia* spp. *B*. *thailandensis* E264 **(A)** or *B*. *pseudomallei* K9264 **(B)** strains were incubated statically in peg-assay 96-well plates as described at 37°C for 48 h. Biofilm formation was assessed by crystal violet staining and measurement of optical density at 550 nm. Student’s *t*-test was performed for each species’ wild-type versus the mutant strain using GraphPad Prism 8.1.2. Error bars represent standard deviation from the mean of seven technical repeats. A representative figure from three independent biological replicates is shown for each species. ***p < 0.001.

In order to characterize the PglL_Burk_ mutants in *B*. *pseudomallei* and *B*. *thailandensis*, we first utilized cell-free *in vitro* based assays to determine phenotypes that may be affected by deletion of the *O*-OTase pathway. Pilin, a reported target for glycosylation by PglL has been shown to promote biofilm formation and twitching-based motility. We examined the ability of the strains to adhere to an abiotic surface in the form of polypropylene pegs at 37°C over 48 h. *Δpg^lL^Bthai* was significantly attenuated in its ability to form biofilms, although it was still capable of adhering to the peg surface (Figure 1A). The *ΔpglL_BPS_* mutant also showed reduced biofilm formation (Figure 1B). These data suggest that PglL supports, but is not essential for biofilm formation in either *Burkholderia* species.

**Figure 2:**
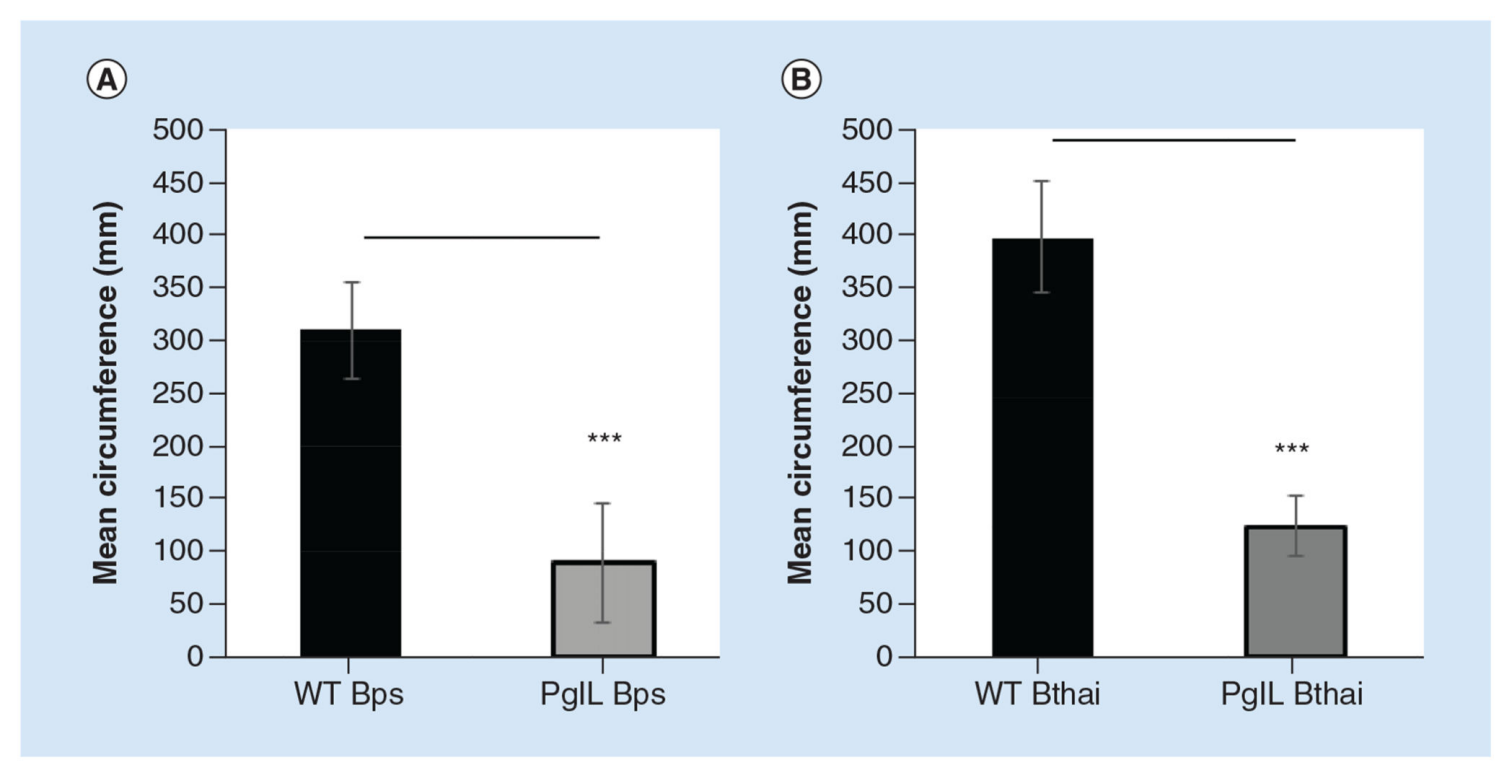
Twitching motility of wild-type and Δ*pglL Burkholderia* spp. *B*. *pseudomallei* K9264 **(A)** or *B*. *thailandensis* E264 **(B)** strains were grown between a plastic-agarose interface at room temperature and resultant mean colony circumference measured. Student’s *t*-test was performed for each species’ wild-type versus the mutant strain using GraphPad Prism 8.1.2. Error bars represent standard deviation from the mean of triplicate technical repeats. A representative figure from three independent biological replicates is shown. ***p < 0.001.

We also assessed twitching motility, by measuring the mean circumference of colonies growing between an agarose and a plastic surface. For both *Burkholderia* spp., the wild-type strains had significantly greater colony size compared with the *ΔpglL* mutant, indicating an important contribution of PglL to twitching motility (Figure 2A & B).

### PglL_BPS_ promotes resistance to cell surface stress

Since PglL may modulate proteins expressed at the cell surface, we examined the sensitivity of the strains to cell surface attack.

**Figure 3:**
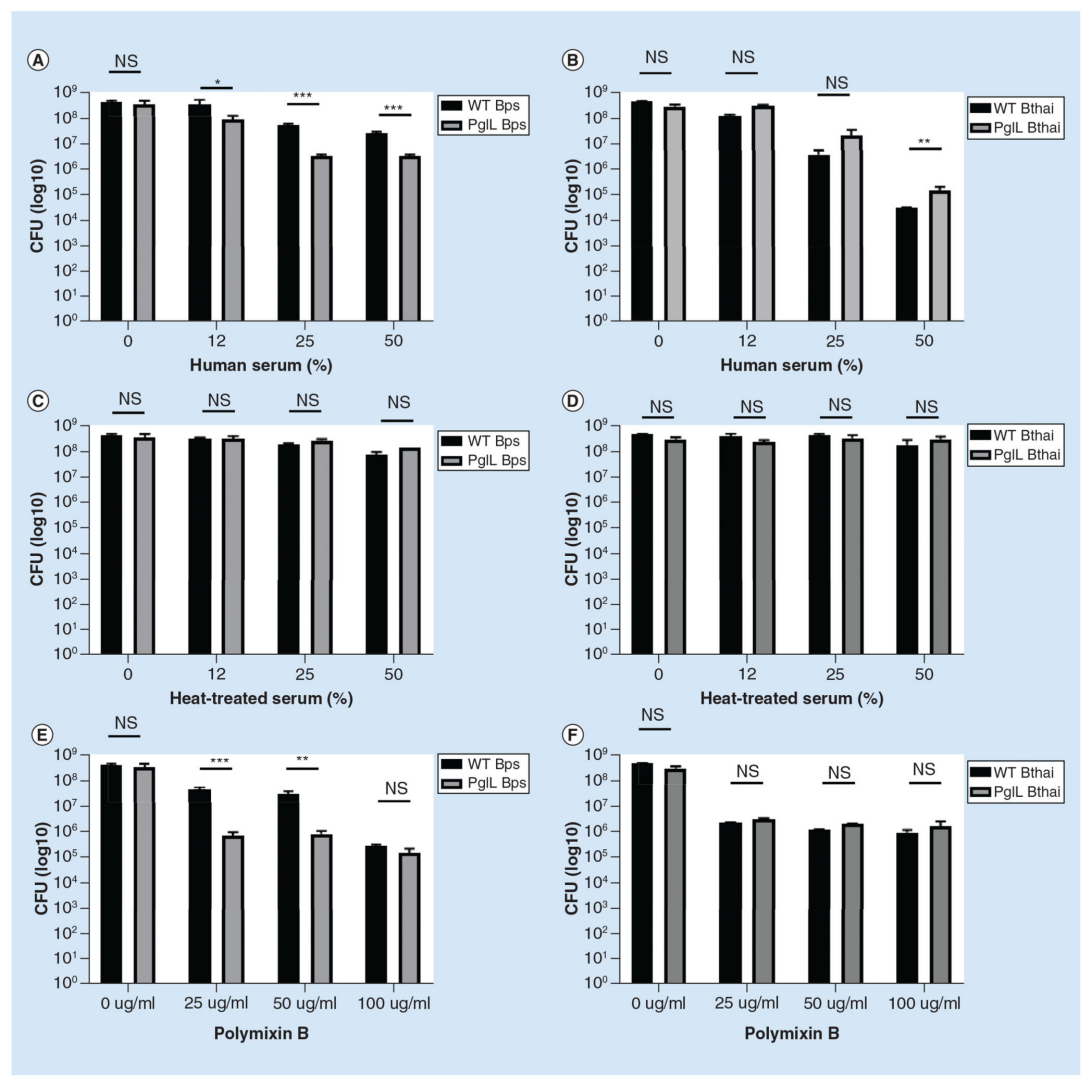
Sensitivity of wild-type and Δ*pglL Burkholderia* spp. to Polymixin B and serum. *B*. *thailandensis* E264 or *B*. *pseudomallei* K9264 strains were incubated with a titration of either human serum **(A & B)**; heat-treated serum **(C & D)**; or polymixin B **(E & F)**. Alternatively, human serum was heat inactivated by treatment at 65°C for 1 h. Student’s *t*-test was performed for each species’ wild-type versus the mutant strain within each condition using GraphPad Prism 8.1.2. Error bars represent standard deviation from the mean of triplicate technical repeats. A representative figure from three independent biological replicates is shown. *p < 0.05; **p < 0.01; ***p < 0.001. CFU: Colony-forming unit.

The Δ*pglL_BPS_* mutant was more sensitive than wild-type to killing mediated by complement proteins in human serum at concentrations above 12% (Figure 3A & B), a conclusion supported by the lack of killing when human serum was heat treated to denature complement in the serum (Figure 3C & D). Interestingly, the wild-type *B*. *thailandensis* strain was more sensitive complement mediated killing than even the Δ*pglL_BPS_* mutant, and deletion of *pg^lL^Bthai* enhanced resistance to attack.

Polymixin B, a cationic peptide, was significantly more toxic to Δ*pglL_BPS_* than to the wild-type strain, while the Δ*pglL_Bthai_* mutant was equally sensitive to polymyxin B as the wild-type (Figure 3E–F). At higher concentrations of polymyxin B, there was no significant difference between the Δ*pglL_BPS_* mutant and wild-type strain.

### PglL_BPS_ is required for efficient cell invasion & intracellular survival

**Figure 4:**
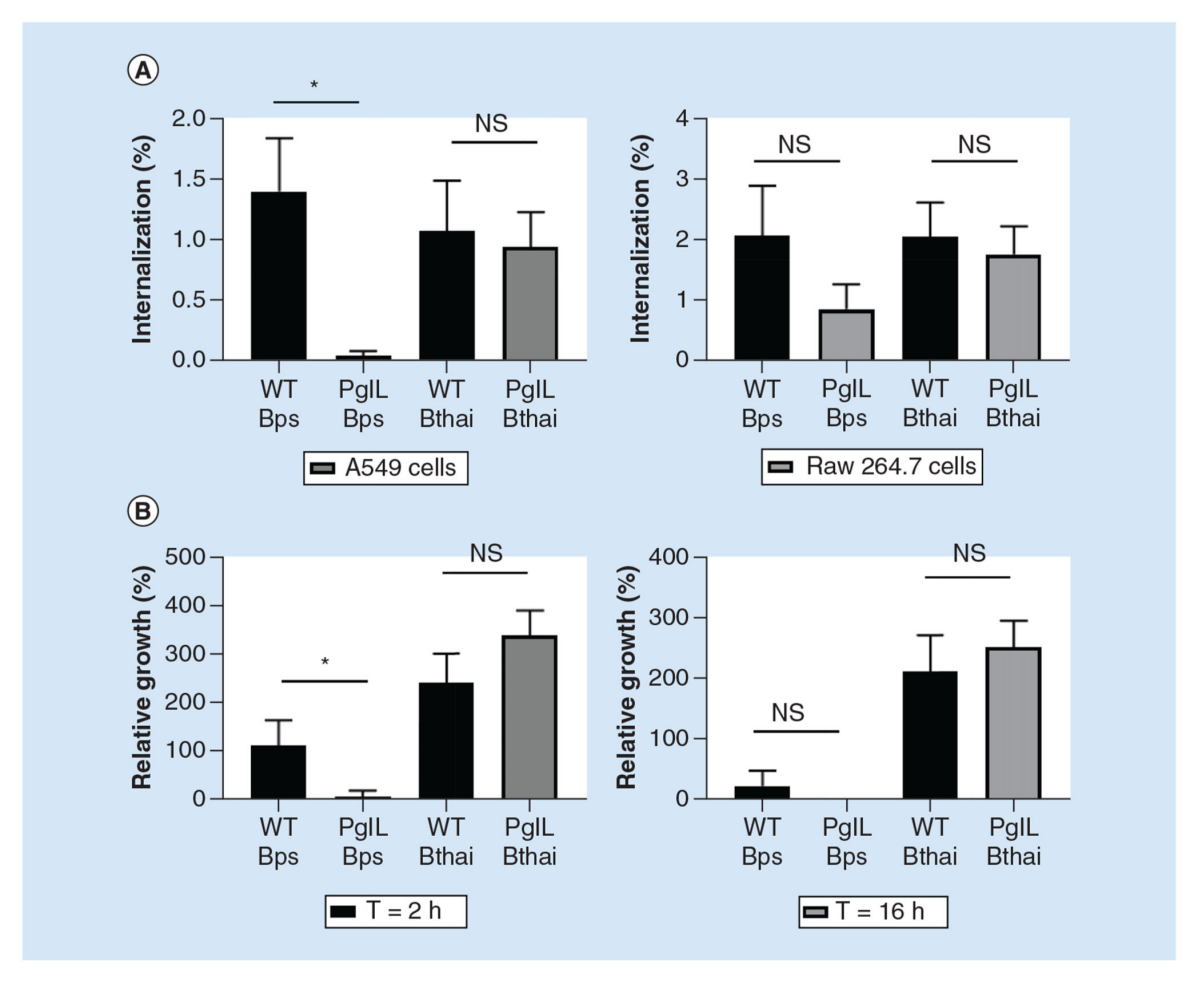
Attachment, uptake and intracellular survival of wild-type and Δ*pglL Burkholderia* spp. *Burkholderia* spp. strains were used to infect either human epithelial A549 cells or murine Raw 264.7 cells at an multiplicity of infection of five for 90 min before washing, lysis and enumeration of bacteria by colony-forming unit assay **(A)**. Raw 264.7 cells were infected for longer time periods as described, with kanamycin used to control extracellular bacterial replication. Values are expressed as proportion of cells versus the infective dose **(A)** or versus 90 min colony-forming unit **(B)**. Student’s *ŕ*-test of wild-type versus mutant within each group was performed using GraphPad Prism 8.1.2. Error bars represent standard deviation from the mean of triplicate technical repeats. A representative figure from three independent biological replicates is shown. **p < 0.05; ***p < 0.001. WT: Wild-type.

Attachment and adherence to surfaces is the first step toward host–cell invasion, and an important aspect of *Burkholderia* spp. virulence. Considering the observed influence of *pglL* on biofilm formation, we wanted to understand whether there would be a similar contribution to eukaryotic cell adhesion. While the Δ*pglL_Bthai_* mutant displayed no distinguishable phenotype compared with wild-type *B*. *thailandensis*, the Δ*pglL_BPS_* mutant was severely impaired in its ability to invade host cells (Figure 4A). This was particularly acute when an A549 epithelial cell monolayer was used, although the mutant was also deficient in RAW 264.7 cell entry. Following uptake, the Δ*pglL_BPS_* mutant was subsequently attenuated in intracellular survival in RAW 264.7 macrophagelike cells compared with wild-type *B*. *pseudomallei* (Figure 4B). Δ*pg^lL^Bthai* was not significantly impaired in its intracellular survival.

### PglL_BPS_ is required for efficient actin tail assembly, cell division & plaque formation in A549 epithelial cell monolayers

**Figure 5:**
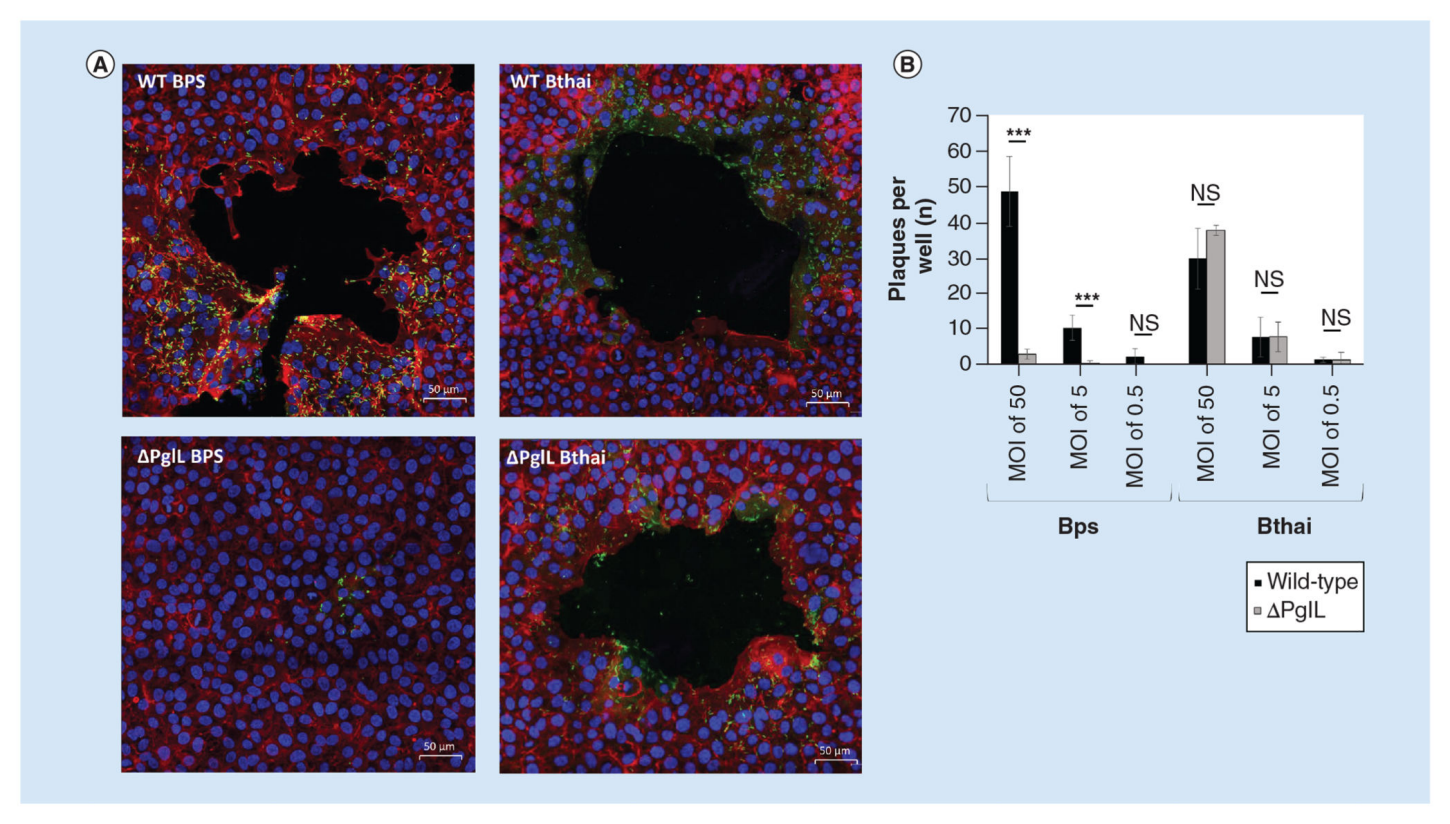
Plaque formation by wild-type and Δ*pglL Burkholderia* spp. In an A549 cell monolayer. *Burkholderia* spp. strains were used to infect a confluent monolayer of A549 cells in chamber slides. After 90 min infection at different multiplicity of infections, cells were incubated in the presence of 100 μg ml^-1^ kanamycin for a further 16 h. Immunofluorescence with confocal microscopy was used to image the monolayers, with nuclei stained with DAPI (blue), host-cell actin cytoskeleton was stained with phalloidin-alexafluor-546 (red) and bacteria visualized using an alexafluor-488 secondary antibody **(A)**. For counting, plaques were visualized using phase-contrast microscopy and the entire well of duplicate wells was used for counting for each condition **(B)**. Student’s *t*-test of wild-type versus mutant was performed within each species and multiplicity of infection group using GraphPad Prism 8.1.2. Error bars represent standard deviation from the mean of duplicate technical repeats. A representative figure from two independent biological replicates is shown. ***p < 0.001. WT: Wild-type.

**Figure 6:**
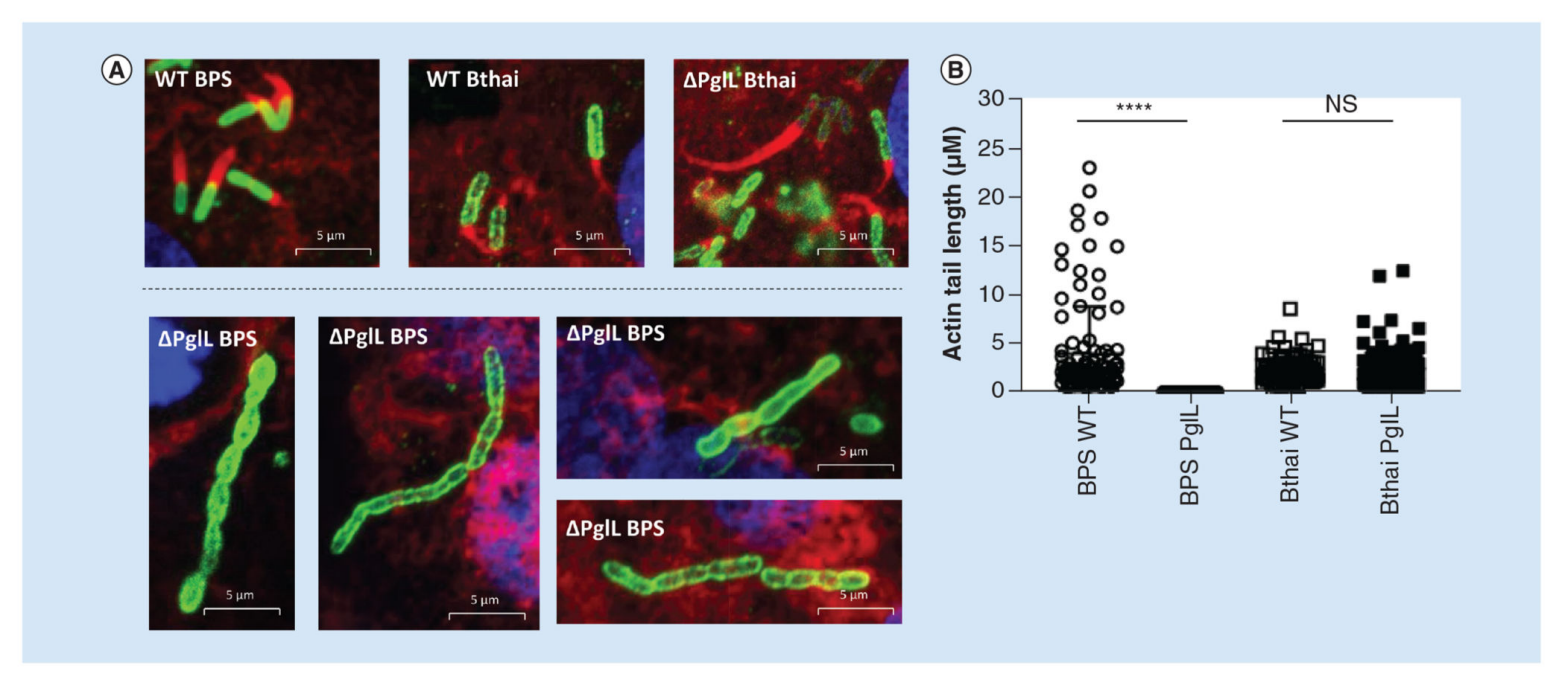
Actin-tail formation by wild-type and Δ*pglL Burkholderia* spp. in an A549 cell monolayer. *Burkholderia* spp. strains were used to infect a confluent monolayer of A549 cells in chamber slides. After 90 min infection at different multiplicity of infections, cells were incubated in the presence of 100 μg ml^-1^ kanamycin for a further 16 h. Immunofluorescence with confocal microscopy was used to image the monolayers, with nuclei stained with DAPI (blue), host-cell actin cytoskeleton was stained with phalloidin-alexafluor-546 (red) and bacteria visualized using an alexafluor-488 secondary antibody **(A)**. To measure actin tails, Zeiss Zen software was used, and the results displayed as a scatter chart **(B)**. Mean lengths were: BPS WT 3916 nm (n = 106); BPS Δ*pglL* 3 nm (n = 43); Bthai WT 2204 nm (n = 86); Bthai Δ*pglL* 2222 nm (n = 112). Statistical difference between mutant and wild-type for each species was determined by Mann–Whitney U test using GraphPad Prism 8.1.2. ****p < 0.0001.

Having established altered host-pathogen interaction in the Δ*pglL_BPS_* mutant, we wanted to further examine its role in virulence by utilizing confocal immunofluorescence to visualize interaction of the bacteria with a confluent A549 cell monolayer. Both the *B*. *pseudomallei* and *B*. *thailandensis* wild-type strains, as well as the Δ*pglL_Bthai_* mutant were capable of inducing multiple large plaques in the monolayer, in an MOI-dependent manner, and could readily polymerise host-cell actin to form characteristic ‘comet tail’ structures (Figure 5A & B). Mean actin tail length generated by wild-type *B*. *thailandensis* was significantly shorter than those generated by wild-type *B*. *pseudomallei* (at 3916 and 2204 nm, respectively; Figure 6B). In the Δ*pglL_BPS_* mutant, actin tail formation was rarely detected, and the mean length of those tails was significantly shorter than in either wild-type species, at just 30 nm.

Plaque formation was not readily apparent when RAW 264.7 macrophages were used (data not shown). Bacteria appeared to be mostly concentrated at the perimeter of the plaques in A549 cells, and many appeared to be in the process of cell division. By contrast, deletion of *pglL* in *B*. *pseudomallei* resulted in significantly reduced and truncated actin tail formation, with severely restricted plaque formation. In agreement with our cell infection assay, very few Δ*pglL_BPS_* bacteria were observed intracellularly after a 16 h infection. Additionally, those bacteria that were present exhibited a severe cell-separation defect to form filamentous chains of bacilli (Figure 6A).

### PglL_BPS_ contributes to inter-species bacterial competition

The observation that the Δ*pglL_BPS_* mutant was unable to efficiently induce actin tails or induce plaque formation suggested a possible role for PglL in Type III and/or Type VI secretion system-mediated virulence. We chose to investigate this aspect further by establishing an *in vitro* assay to study inter-species bacterial competition; when cocultured on an agarose surface, competition between bacteria is greatly enhanced by the presence of a functional Type VI secretion system.

**Figure 7:**
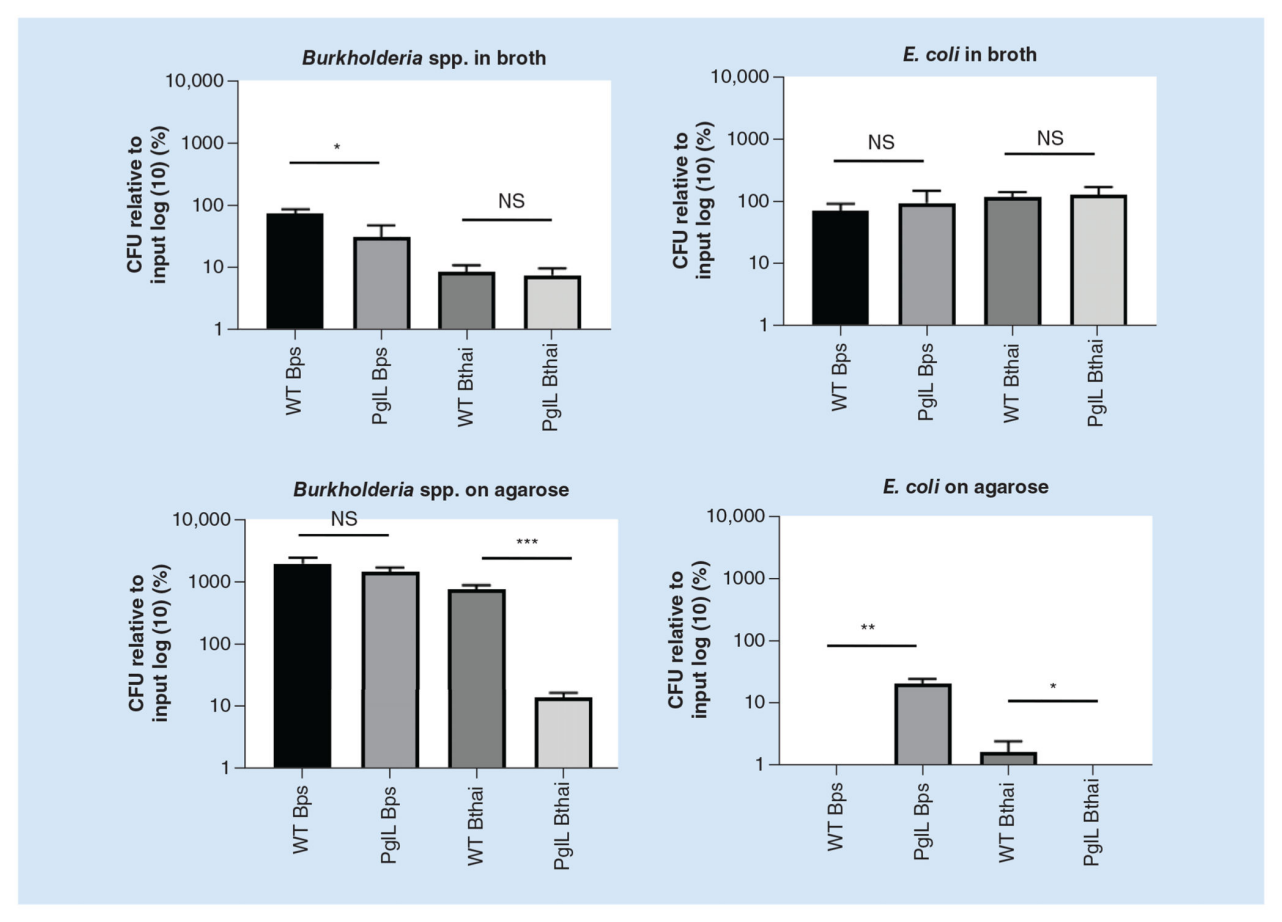
Competition assay between wild-type or Δ*pglL Burkholderia* spp. and *E*. *coli*. Burkholderia spp. strains were cocultured with *E*. *coli*_pcDNA 3.3 TOPO_*LacZ* at identical optical densities for 5 h in either LB broth or spotted onto LB agar, as indicated. Bacteria were enumerated by colony-forming unit assay on agar plates coated with X-gal for distinguishing bacterial species by blue/white screening. Student’s *t*-test was performed for each species’ wild-type versus the mutant strain within each condition. Using GraphPad Prism 8.1.2. Error bars represent standard deviation from the mean of triplicate technical repeats. A representative figure from three independent biological replicates is shown. *p < 0.05; ***p < 0.001. CFU: Colony-forming unit; WT: Wild-type.

There was no significant difference in the CFU of *E*. *coli* when cocultured in broth with either the *pglL* mutant (of either species) or the wild-type (Figure 7). However, on agarose, there were significantly fewer *E*. *coli* when cocultured with wild-type *B*. *pseudomallei* than with the *pglL* mutant, despite there being no significant difference in the CFU of *Burkholderia* spp. This is suggestive of a defect in interbacterial competition in the *pglL* mutant in

*B*. *pseudomallei*. For *B*. *thailandensis*, there were significantly fewer *pglL* mutant and *E*. *coli* bacilli cocultured on agarose.

### PglL_BURK_ is a key regulator of virulence *in vivo*

**Figure 8:**
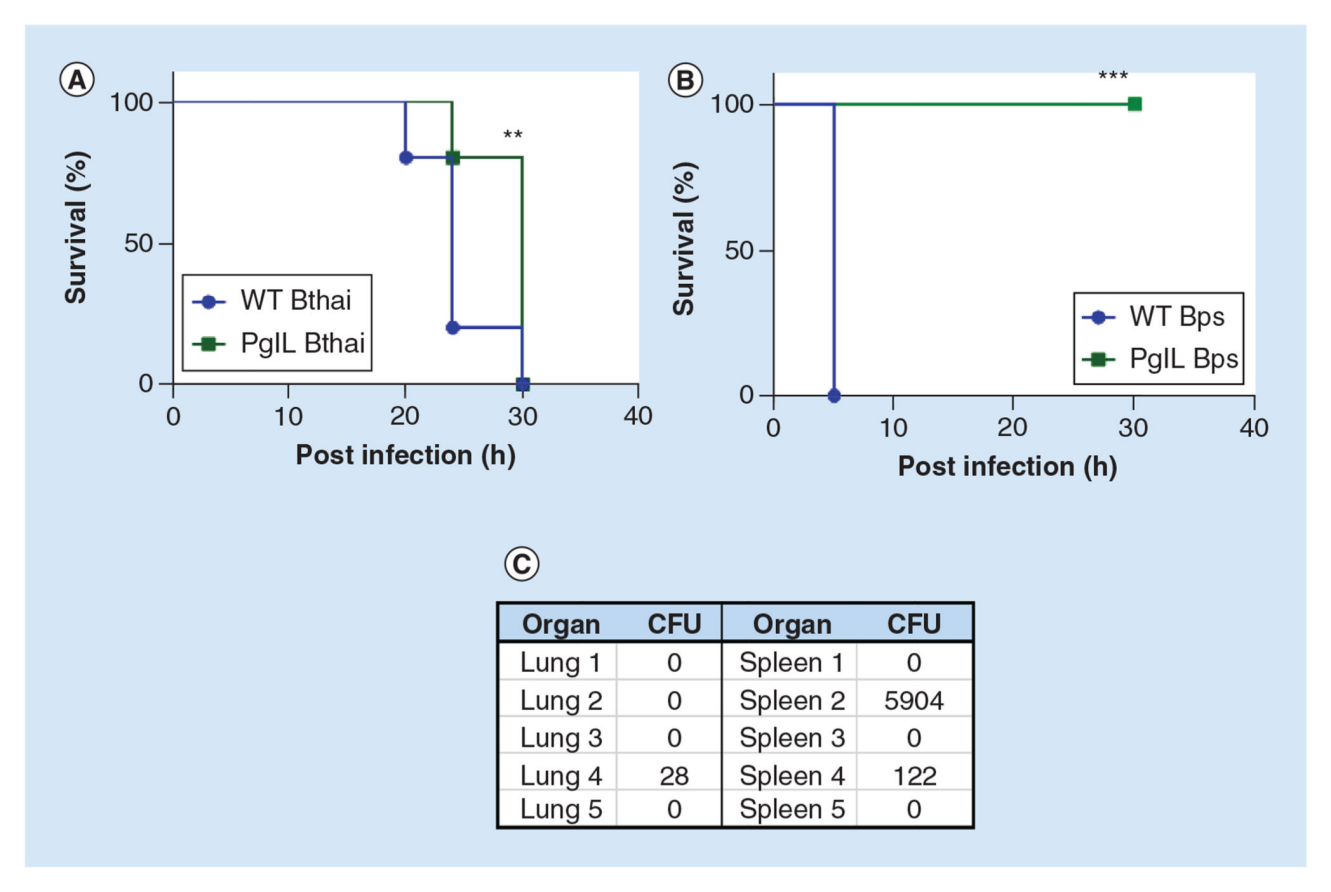
*In vivo* virulence of wild-type (WT) and Δ*pglL Burkholderia* spp. *G*. *mellonella* larvae (n = 10 per group) were infected with 1000 colony-forming unit Bthai WT or Bthai Δ*pglL* or inoculated with PBS alone. After 36 h, difference between WT E264 survival was compared with mutant pglL **(A)**. Female BALB/C mice (n = 5 per group) were infected intranasally with approximately 1000 colony-forming unit either WT BPS K92643 or BPS Δ*pglL* in sterile saline **(B)**. Surviving mice were culled at day 30 post infection and total bacterial colony-forming unit in lungs and spleen enumerated **(C)**. **p < 0.05 Log-rank Mantel-Cox and Gehan-Breslow-Wilcoxon test; ***p < 0.01 Log-rank Mantel-Cox and Gehan-Breslow-Wilcoxon test. CFU: Colony-forming unit; WT: Wild-type.

Having observed several effects of *pglL* deletion *in vitro*, we hypothesized that the mutants might be attenuated in an *in vivo* model of infection. Since *B*. *thailandensis* is avirulent in the murine model, we utilized a previously validated approach of infection using *G. mellonella* wax-moth larvae as the host organism. Both wild-type *B*. *thailandensis* and Δ*pglL_Bthai_* were able to kill all *G*. *mellonella* within 30 h, but infection with the mutant was able to significantly extend the time until death for the majority of the larvae (Figure 8A).

Using a murine model of acute melioidosis [26], we identified that the Δ*pglL_BPS_* mutant was significantly attenuated (Figure 8B). When infected intranasally with approximately 1000 CFU of wild-type *B*. *pseudomallei*, all mice developed clinical signs consistent with melioidosis infection and were culled after 4 days. By contrast, all mice infected with the Δ*pglL_BPS_* strain were active and healthy at 30 days, when the experiment was terminated. To establish whether or not the animals had eradicated the infection, we assessed the organ load of *B*. *pseudomallei* in both lungs and spleens (Figure 8C). Our data show that three out of five mice achieved sterility within the time limit of the experiment, and had resolved the infection completely. The remaining two mice both retained Δ*pglL_BPS_* bacilli in the spleen, and one mouse also retained viable bacteria in the lung despite being clinically healthy.

### Vaccination with *B. pseudomallei ΔpglL* enables the murine host to mount an improved protective immune response against subsequent lethal-dose acute *B*. *pseudomallei* wild-type infection

**Figure 9:**
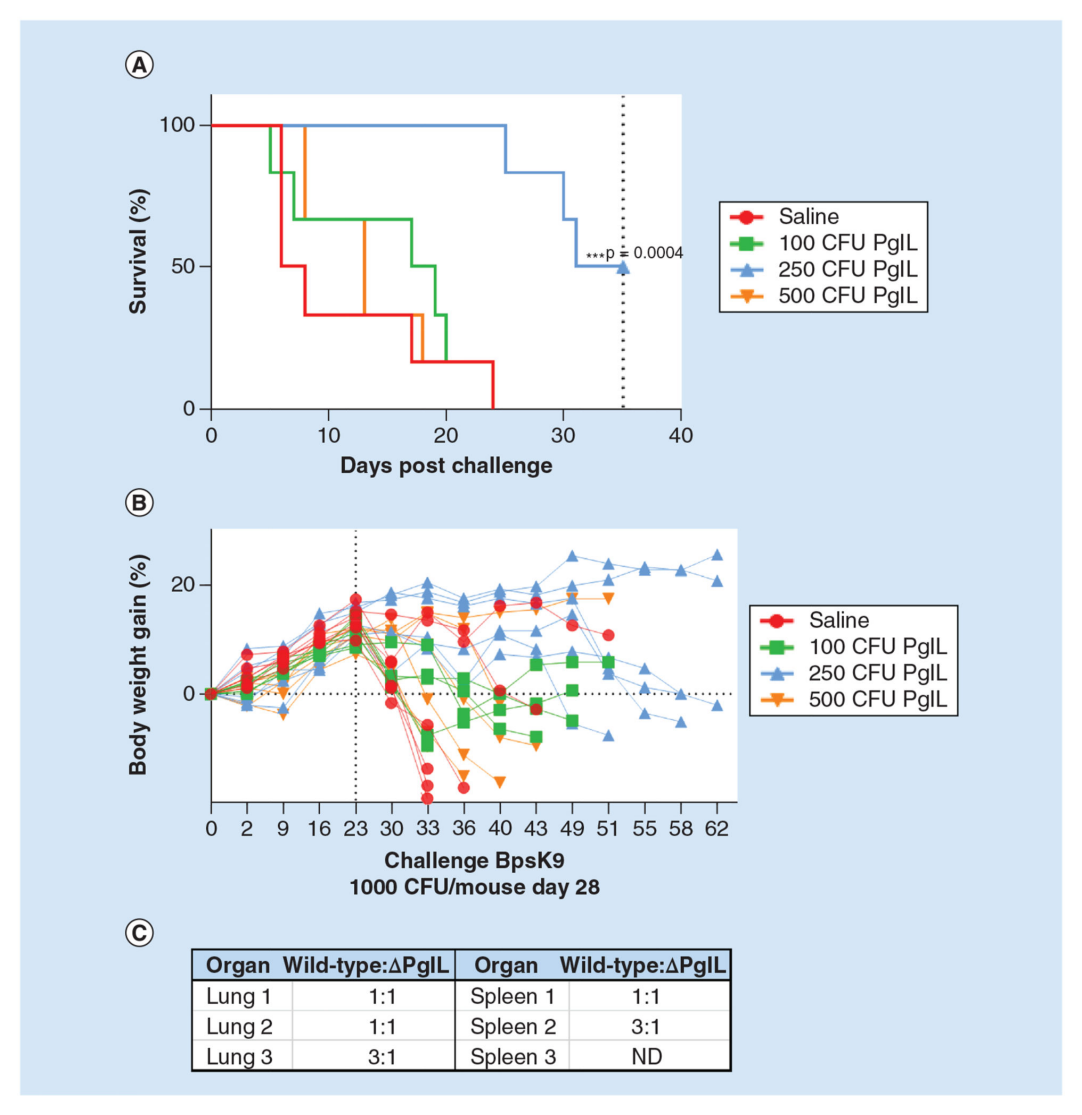
Vaccination of female BALB/C mice with Δ*pglL B. pseudomallei* and challenge with wild-type BPS K9264. Female BALB/C mice (n = 6 per group) were infected intranasally with different colony-forming unit of BPS Δ*pglL* as indicated, and after 28 days, challenged intranasally with wild-type BPS K9264. Survival **(A)** and body weight **(B)** were monitored up to 68 days post vaccination. At 68 days, surviving mice (from the 250 CFU vaccinated group) were culled and strains of organ-resident bacteria identified by polymerase chain reaction for the *pglL* gene to establish the ratio of wild-type (challenge strain) to Δ*pglL* (vaccine strain) colonies **(C)**. ***p < 0.01 Log-rank Mantel-Cox and Gehan-Breslow-Wilcoxon test. CFU: Colony-forming unit; ND: Excluded due to splenomegaly.

Since we demonstrated that Δ*pglL_BPS_* was severely attenuated in mice, and the majority cleared the infection from the host, we were interested to establish whether it may provide proof of concept as a potential candidate for vaccination against *B*. *pseudomallei* infection. To attempt a scenario whereby the host was able to reproducibly clear the Δ*pglL_BPS_* infection before challenge with wild-type *B*. *pseudomallei*, we experimented by reducing the inoculum of the mutant to 500, 250 and 100 CFU for the vaccination experiment. Interestingly, we observed an inoculum dose-dependent protective effect of vaccination with Δ*pglL_BPS_* (Figure 9A). While mice that received the lowest and highest dose of the mutant did not survive significantly longer than the saline-treated control group, those that received a moderate dose of 250 CFU Δ*pglL_BPS_* had significantly extended time until death. On average, the mice lost weight following challenge with wild-type *B*. *pseudomallei*; however mice vaccinated with 250 CFU Δ*pglL_BPS_* showed either no or limited weight loss, reflecting their reduced disease severity (Figure 9B).

Surviving mice were culled at 35 days post-challenge so that organ bacterial burden could be examined. By using PCR to distinguish the strains of *B*. *pseudomallei*, we found that all surviving mice harbored a mixed infection of both Δ*pglL_BPS_* and wild-type *B*. *pseudomallei* (Figure 9C), consistent with our previous finding that mutant strain could not be consistently cleared by the host. The numbers of mutant to wild-type bacteria were approximately equal, and in some cases the wild-type strain was the most prevalent.

## Discussion

By creating isogenic marker-less deletion mutants of the *pglL* gene in the avirulent species, *B*. *thailandensis* and in the human pathogen, *B*. *pseudomallei*, we were able to characterize its contribution to fitness and virulence among the *Burkholderiaceae*.

The Δ*pglL* mutant in both species appeared to have pleiotropic effects. Loss of *pglL* negatively impacted biofilm formation and twitching motility in both species. Considering the close proximity of *pglL* to *pilA*, and that this is a reported target for glycosylation by *pglL* in other species, our data are consistent with a role for PglL in pilin-related function. While *pglL* significantly contributed to biofilm formation on abiotic surfaces, it was not completely abolished in the mutant strains. PilA-independent adherence in *B*. *pseudomallei* K96243 has been suggested by Boddey *et al*. [27]. PilA is also implicated in supporting twitching motility [28], and our data suggest that *pglL* supports this phenotype in both species. These findings are interesting in the context of the work of Lithgow *et al*. and Mohamed *et al*., who did not identify pilin as one of the glycoproteins dependant on *pglL*, although there may be technical challenges in isolating these fragile structures [11,14].

Besides these two shared phenotypes, deletion of *pglL* resulted in species-restricted outcomes. The efficient attachment to and invasion of host cells by *B*. *pseudomallei* has been shown to rely on type IV pilin [29], as well as a functional type III secretion system (T3SS) [30]. We found that the PglL_BPS_, but not the _Pg_lL_BtBai_ mutant was defective in cell invasion in A549 epithelial-like cells. The fact that the phenotype was more apparent with A549 cells, which are non-phagocytic, than in RAW 264.7 macrophage-like cells implies that the PglL_BPS_ is specifically involved in attachment and invasion rather than via uptake mediated by ligation of host pattern recognition receptors.

The Δ*pglL_BPS_* mutant also demonstrated attenuated intracellular survival after 2 h in macrophage-like cells, whereas the *pg^lL^Bthai* mutant did not. Here also, the T3SS has been shown to be essential, as it is required for evasion of phagosomal [31] and autophagosomal [32] maturation. It was interesting to observe that more wild-type *B*. *thailandensis* bacteria were present in infected cells after 16 h than wild-type *B*. *pseudomallei*, the more virulent species, however others have reported similar findings [33]. The important role of the T3SS in cellular invasion and early intracellular survival of *B*. *pseudomallei* is well established – our findings now support that *pglL* has similar relevance. Further work is required to establish whether there is a direct connection between secretion system activity and *O*-linked glycosylation, or whether they function as independent virulence factors.

By utilizing immunofluorescence, we were able to visualize additional aspects of infection that can result in tissue pathology in the host. The primary antibody that we used was raised against *B*. *pseudomallei* K96243 strain, which cross-reacted with *B*. *thailandensis* E264 - suggesting the conserved Type II O-antigen [34] may be the target epitope. Only in the Δ*pglL_BPS_* mutant, we observed reduced intracellular survival, actin-tail defects, abrogated plaque formation and a cell division deformity. Actin-tail formation was less efficient in wild-type *B*. *thailandensis* than *B*. *pseudomallei*, supporting published observations that *B*. *thailandensis* utilises an alternative mechanism to induce host actin polymerisation [35]. In both species however, the upstream events rely on effectors of the T3SS to facilitate phagosomal escape, before the autotransported protein, BimA, with BimC, recruit the necessary actin polymerisation complexes at the poles of the bacillus [36]. Actin-tail formation in the Δ*pglL_BPS_* mutant was very rare, and when it occurred, the actin-tail length was significantly truncated. This suggests that at least some of the Δ*pglL_BPS_* bacilli could escape the phagosomal vacuole, but were thereafter impaired in their ability to polymerise actin.

Cell-to-cell spread of *B*. *pseudomallei*, cell fusion, giant-cell formation and ultimately, plaque formation are dependent on the type VI secretion system (T6SS) [37]. T6SS are fairly widespread among Gram-negative bacteria, having been identified in around one quarter of sequenced isolates [38,39]. In the extracellular environment, they facilitate interspecies bacterial competition [40] while in the intracellular compartment, they have been reported as having a role in phagosomal escape and in cytoplasmic replication [41–43]. Lack of plaque formation by the Δ*pglL_BPS_* mutant could have been simply due to its reduced ability to survive intracellularly, we therefore conducted an *in vitro* competition assay to further explore a role for PglL in T6SS function.

The T6SS depends upon very close proximity for the effective injection of toxins into prey species, and so its activity is restricted to solid surface or biofilms [44]. Therefore in our experiment when *E*. *coli* was cocultured with *B*. *pseudomallei* in LB broth, when growth was planktonic, the *E*. *coli* could evade T6SS-mediated killing; we found no significant difference in *E*. *coli* CFU in coculture with either Δ*pglL_BPS_* or wild-type in this condition. However, on an agarose surface, *Burkholderia* spp. could effectively kill the *E*. *coli*, pointing to an active T6SS. Deletion of *pglL_BPS_* significantly perturbed this ability, with approximately 2.5 logs greater *E*. *coli* CFU after coculture compared with the wild-type. Since the CFU of the *Burkholderia* wild-type and mutant was not significantly different on agarose, our results suggest the defect lies in attenuated bactericidal activity, and not a general growth defect in the mutant.

We also observed distinct morphological differences relating to aberrant cell division between the Δ*pglL_BPS_* mutant and the wild-type strain in A549 cells. Lack of *pglL* might affect cell wall integrity in the mutant in numerous ways. For example, through altered expression and/or trafficking of cell surface lectins [16] and glycoproteins that might otherwise accumulate in the periplasm. Furthermore, since PglL transfers oligosaccharide from the UndPP carrier [45], its deletion may prevent recycling of UndPP for use as a carrier of peptidoglycan, lipopolysaccharide or capsule precursors, stalling the biosynthesis of these structures. It should be noted however, that a *pglL* mutant in *B. cenocepacia* was recently shown to have no defect in polymeric O-antigen biosynthesis [14]. Non-specific perturbation of the cell wall could be another mechanism behind the increased sensitivity of the Δ*pglL_BPS_* mutant to polymixin B and complement, both of which assemble in the outer membrane.

Cell division is regulated by the assembly of FtsZ subunits at the inner membrane around the circumference of the bacteria. One suggested mechanism is that this ring constricts, pulling the peptidoglycan-bound outermembrane inwards, eventually allowing budding of the daughter cell [46]. Possible disruption of the cell wall in the Δ*pglL_BPS_* mutant may prevent the normal function of the FtsZ ring. Interestingly, mutation of *ftsZ* causes a similar filamentous phenotype to the one we observed. Finally, several glycoproteins have been linked with a role in cell division [47], including FtsZ itself [12], and some of these may be substrates for PglL in *B*. *pseudomallei*, as they have been found to be in *B. cenocepacia* [11].

Collectively, our *in vitro* data account for the remarkable attenuation of the Δ*pglL_BPS_* mutant in the murine model of acute melioidosis. Characteristically for *B*. *pseudomallei*, even in this severely weakened strain, some of the infected mice still harbored bacilli in their lungs and spleen despite showing no clinical signs of disease, indicating that the bacteria could still disseminate from the site of infection and persist in the host. It is notable that in a similar study, in which the *pilA* gene was deleted in *B*. *pseudomallei* K96243, attenuation was observed, but not to the same extent as with deletion of *pglL* [29]. This, along with our own findings presented here, point to the broad contribution *pglL* makes to general cell fitness and virulence beyond a more limited ability to only glycosylate type IV pilin.

There are numerous reasons why PglL may function differently in *B*. *pseudomallei* compared with *B*. *thailandensis*, and it may depend on how PglL interacts with some of the distinguishing features between the species. For example, *B*. *thailandensis* E264 strain naturally possesses a truncated exopolysaccharide capsule compared with *B*. *pseudomallei* K9264, and some strains lack it entirely [48]. This is likely to account for the heightened complement sensitivity of wild-type *B*. *thailandensis* that we observed compared with wild-type *B*. *pseudomallei* [49]. Also, only *B*. *pseudomallei* expresses Type 1 O-antigen in its cell wall [34]. Also, although *B*. *thailandensis* possesses orthologous secretion systems to *B*. *pseudomallei* [37,50,51], they are not completely conserved either: while *B*. *pseudomallei* has three T3SS, and T3SS-3 is almost identical in *B*. *thailandensis*, T3SS-2 is more dissimilar and T3SS-1 is absent [52].

Although their sequences are well conserved at the amino-acid level, evidence to date suggests that PglL glycosylates different peptides in either species [14]. In *B*. *pseudomallei*, glycosylation of ecotin appears to depend upon PglL, which may in turn support its function as a protease inhibitor that can modulate neutrophil activity [53]. BPSL3095 was identified as glycosylated in a PglL-dependent manner by Mohamed *et al*. This is a putative membrane protein that shares ~90% nucleic acid sequence identity over ~50% coverage with some HCP, the T6SS components that extend through the capsule into the extracellular environment [54]. Although there is so far no direct evidence linking PglL with glycosylation of HCP, they do possess numerous serine residues in low complexity regions that suggest they may be permissive substrates. Interestingly, *N*-linked glycosylation of secretion system components has been reported [55] and suggested to aid their mechanical stability [56,57]. Cross-linking of T6SS components with PGN and capsular polysaccharide might support their assembly within the capsule that might otherwise present a barrier [58–61].

Having demonstrated the critical role of PglL in *B*. *pseudomallei* virulence, we also wanted to assess whether in principal, attenuation would allow the infected host to mount a protective immunological response against challenge with fully virulent wild-type *B*. *pseudomallei*. We found an interesting, dose-dependent effect whereby too low a dose of *Δpg^lL^BPS* in the inoculum was not sufficient to induce significant protection, and too high a dose resulted in co-infection with both vaccine and challenge strains; a moderate dose of *Δpg^lL^BPS* did result in significant protection against acute melioidosis. Since sterilising immunity was not achieved in all mice, it would be interesting to see the effect of a vaccine comprised of deletions at multiple loci, each targeting distinct aspects of *B*. *pseudomallei* virulence. For example, the *texR* mutant (intracellular growth) [62] and the 2D2 mutant (capsular polysaccharide) [63] have both been shown to attenuate *B*. *pseudomallei*, and target post-transcriptional regulation and branched chain amino acid biosynthesis, respectively. This multi-target approach might make a live-attenuated vaccine against *B*. *pseudomallei* with sterilizing immunity a reality. In addition, given pleiotropic effect of PglL on virulence, inhibiting this enzyme may be a novel antibiotic target for this naturally multi-antimicrobial resistant pathogen. Our results are particularly interesting in the context of the natural response to *B*. *pseudomallei* infection in humans, whereby patient sera frequently cross-react with *Burkholderia* spp. *O*-glycan, but not with the non-glycosylated version of the peptide [14]. Combined with our results, this suggests a paradigm whereby *O*-glycosylation generates products that are at once immunogenic and required for virulence.

## Conclusion

The data presented here represent the first detailed phenotypic characterization of a Δ*pglL* mutant in *B*. *pseudomallei* with the inclusion of a murine model of melioidosis. We also show that PglL has a differing contribution to virulence in different *Burkholderia* species. Our findings support previous observations in other species that PglL can contribute to biofilm formation and twitching motility, and discover a new role that influences some secretion system-dependent phenotypes in *B*. *pseudomallei*, but not in *B*. *thailandensis*. Finally, our work demonstrates a proof of principle that abrogation of protein glycosylation is a valid strategy for the generation of attenuated strains with the potential for development at a vaccine against melioidosis.

## Supplementary Material

supplementary and Figs Supplementary data
To view the supplementary data that accompany this paper please visit the journal website at: www.futuremedicine.com/doi/suppl/10.2217/fmb-2019-0165

## Summary points

- *Burkholderia pseudomallei* is a highly virulent opportunistic pathogen of humans, difficult to diagnose and treat, with no licensed vaccine.
- *O*-linked OTase glycosylation contribution to virulence has not been explored in this species.
- We delete the *pglL* gene in a virulent and related avirulent species *(Burkholderia pseudomallei* and *Burkholderia thailandensis)*.
- The mutant strains are both defective in twitching motility and biofilm formation.
- Only the *B. pseudomallei pglL* mutant shows a defect in traits that relate to secretion-system mediated virulence.
- The *B. pseudomallei pglL* mutant is highly attenuated in an acute model of melioidosis, and moreover can induce a protective immunological response to subsequent challenge with virulent wild-type *B*. *pseudomallei*.
- Deletion of *pglL* is likely to have pleiotropic effects relating to loss of *O*-OTase function, and possibly secondary effects relating to disruption of other pathways that share aspects of the *pglL* glycosylation pathway.
