# Supplementary material for: Virulence of the emerging pathogen, Burkholderia pseudomallei, depends upon the O-linked oligosaccharyltransferase, PglL
Source: Future Microbiol. Author manuscript; Available in PMC 2021 Jun 17. (PMC7611010; doi:10.2217/fmb-2019-0165)
Supplement: supplementary and Figs [file EMS127599-supplement-supplementary_and_Figs.pdf]

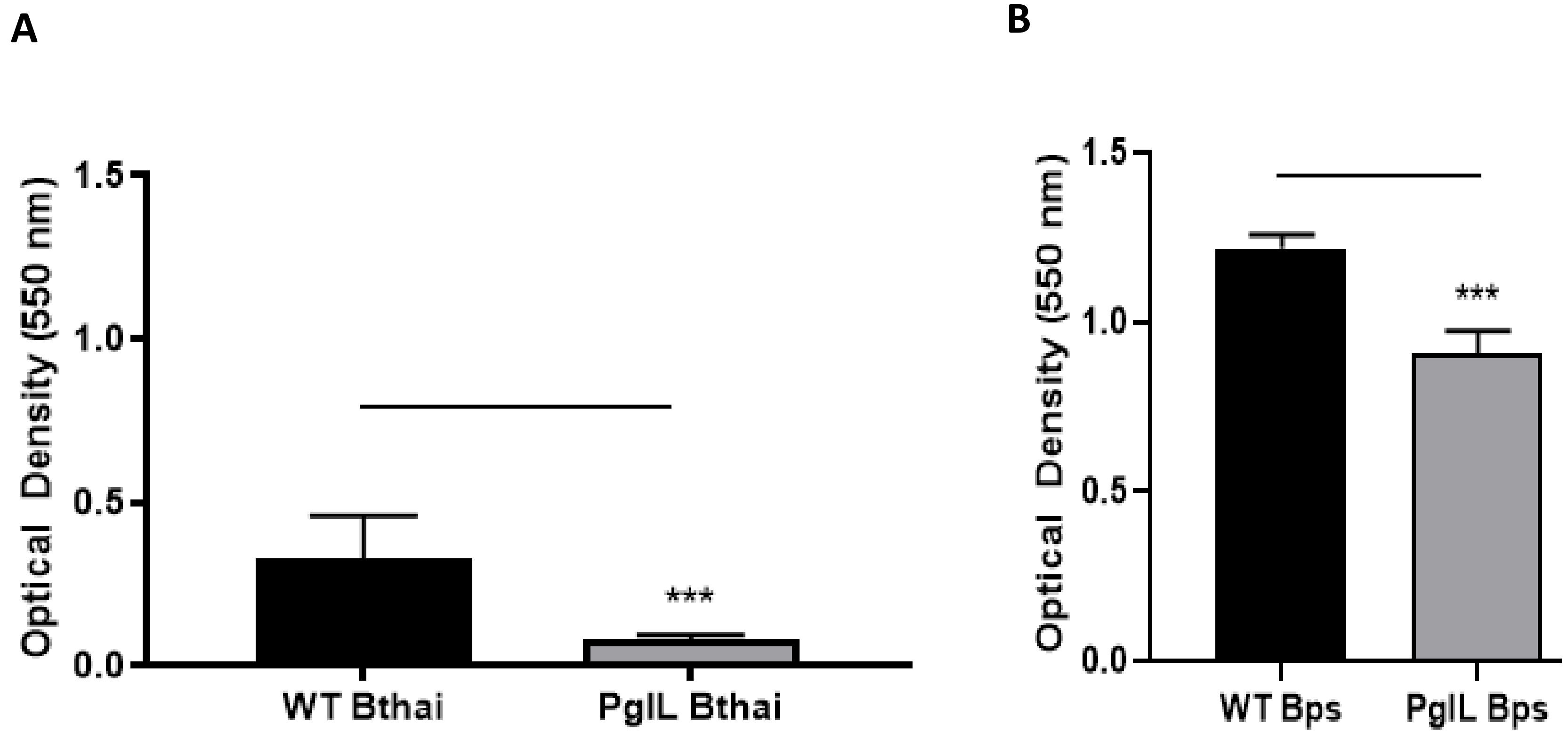

Figure 1: Biofilm Formation in Wildtype and Δ*pglL Burkholderia spp.* *B. thailandensis* E264 (**A**) or *B. pseudomallei* K9264 (**B**) strains were incubated statically in peg-assay 96-well plates as described at 37 °C for 48 hours. Biofilm formation was assessed by crystal violet staining and measurement of optical density at 550 nm. Student's *t*-test was performed for each species' wildtype versus the mutant strain (*** = $p<0.001$) using GraphPad Prism 8.1.2. Error bars represent standard deviation from the mean of seven technical repeats. A representative figure from three independent biological replicates is shown for each species.

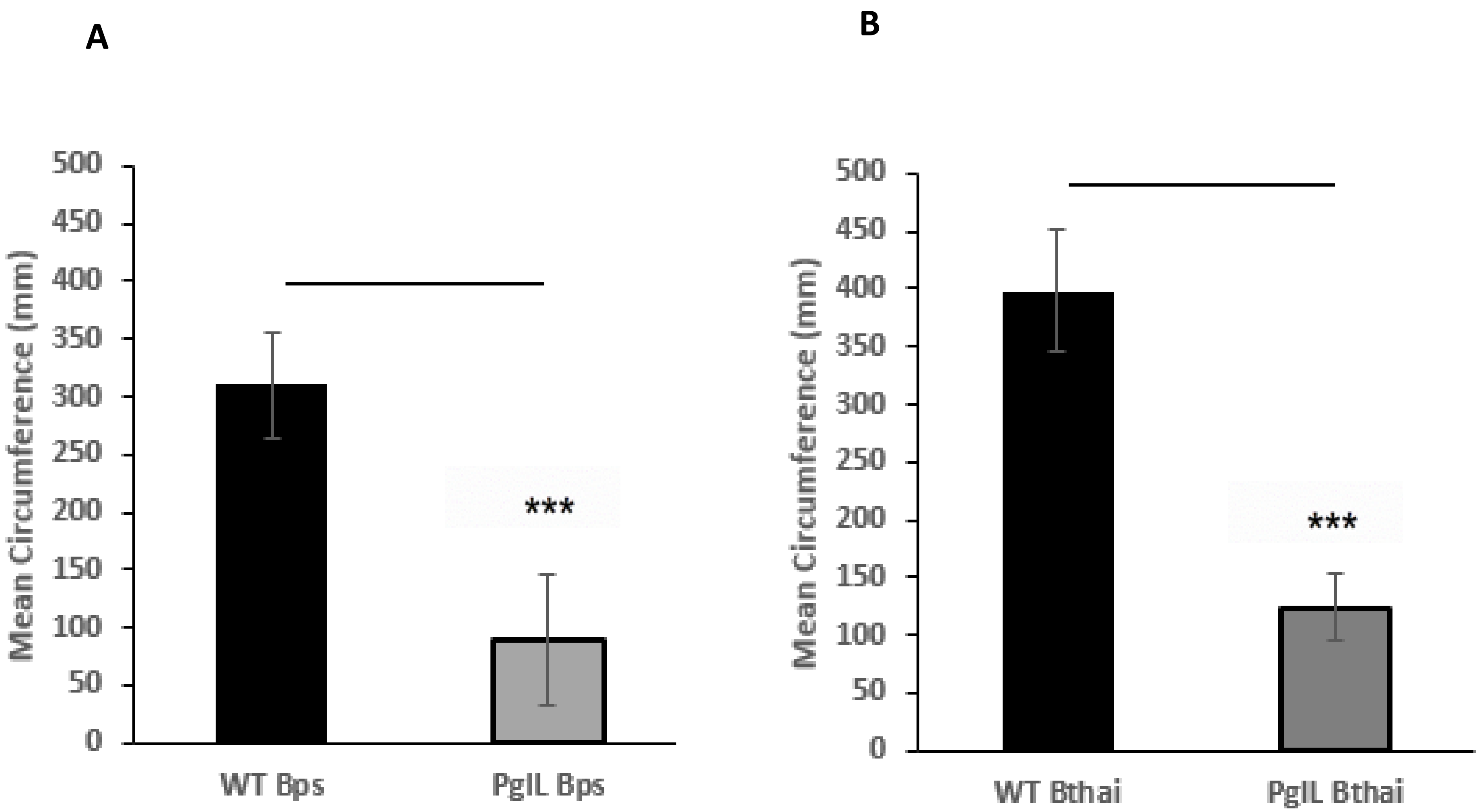

Figure 2: Twitching Motility of Wildtype and Δ*pglL Burkholderia spp.* *B. pseudomallei* K9264 (**A**) or *B. thailandensis* E264 (**B**) strains were grown between a plastic-agarose interface at room temperature and resultant mean colony circumference measured. Student's *t*-test was performed for each species' wildtype versus the mutant strain (*** = $p<0.001$) using GraphPad Prism 8.1.2. Error bars represent standard deviation from the mean of triplicate technical repeats. A representative figure from three independent biological replicates is shown.

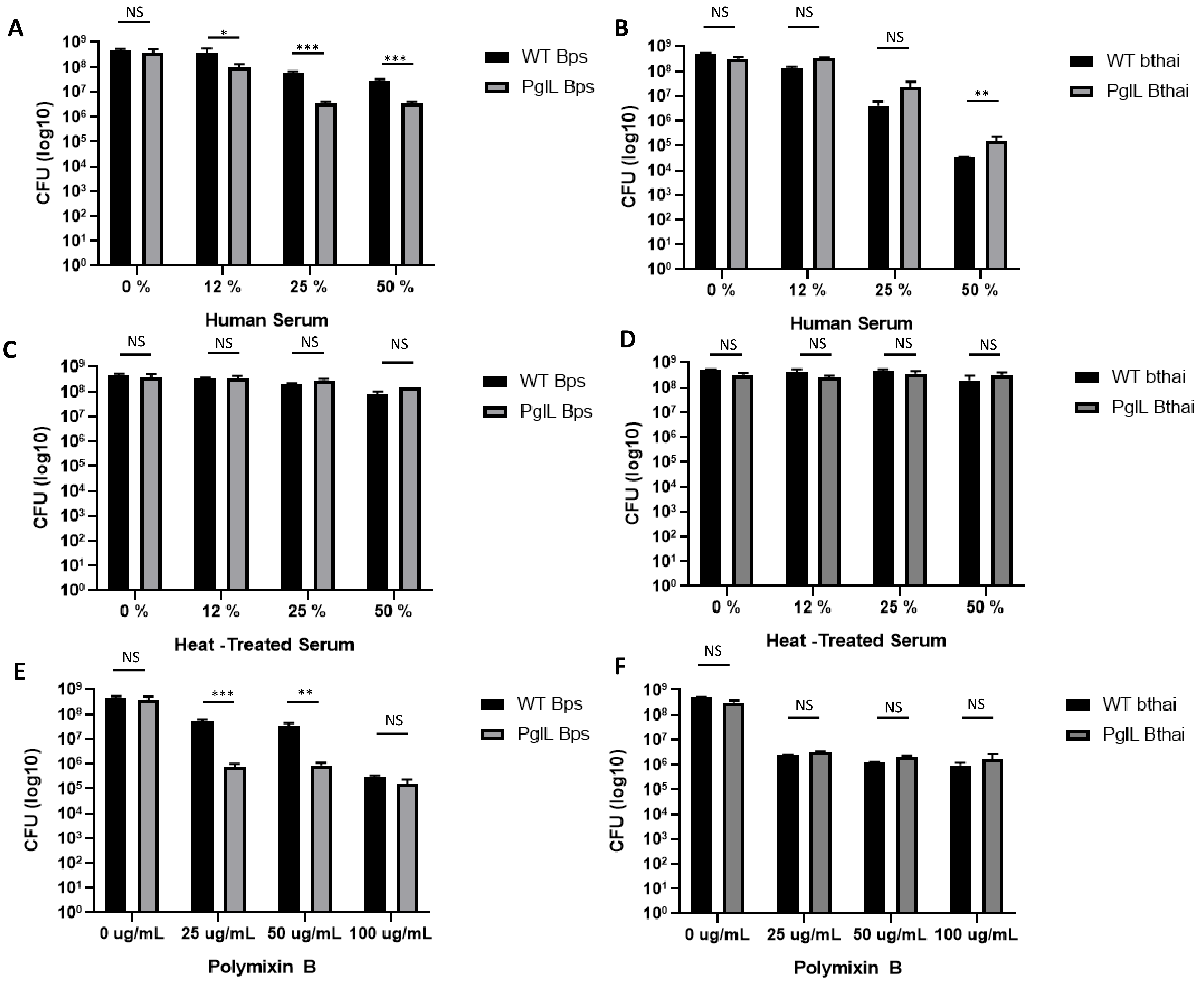

Figure 3: Sensitivity of Wildtype and Δ*pglL Burkholderia spp.* to Polymixin B and Serum. *B. thailandensis* E264 or *B. pseudomallei* K9264 (BPS) strains were incubated with a titration of either human serum (**A+B**); heat-treated serum (**C+D**); or polymixin B (**E+F**). Alternatively, human serum was heat inactivated by treatment at 65 ^{o}C for one hour. Student's *t*-test was performed for each species' wildtype versus the mutant strain within each condition (* = p<0.05; **=p<0.01; ***=p<0.001) using GraphPad Prism 8.1.2. Error bars represent standard deviation from the mean of triplicate technical repeats. A representative figure from three independent biological replicates is shown.

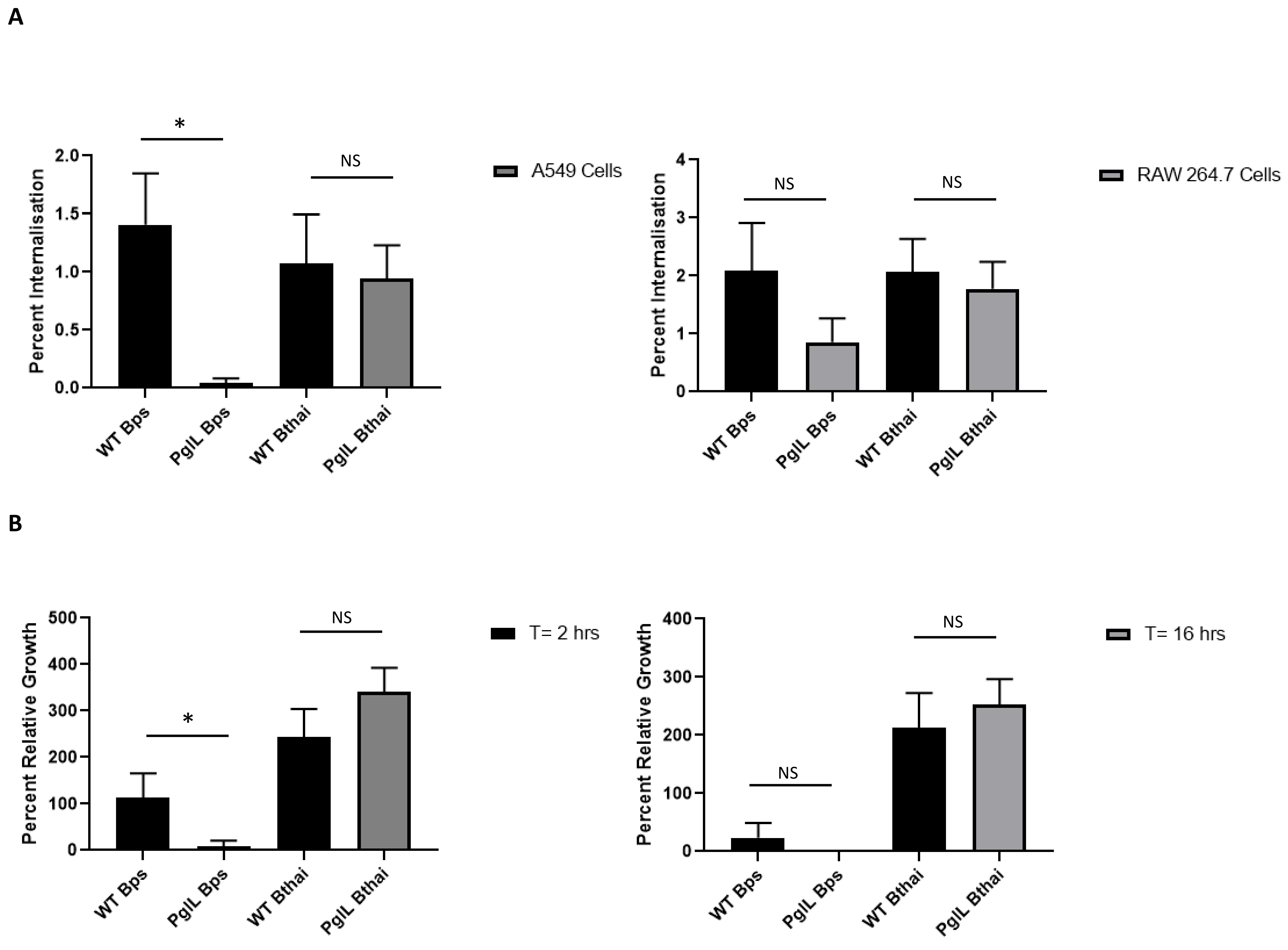

Figure 4: Attachment, Uptake and Intracellular Survival of Wildtype and Δ*pglL Burkholderia spp.*. *Burkholderia spp.* strains were used to infect either human epithelial A549 cells or murine Raw 264.7 cells at an MOI of five for 90 mins before washing, lysis and enumeration of bacteria by CFU assay (**A**). Raw 264.7 cells were infected for longer time periods as described, with kanamycin used to control extracellular bacterial replication. Values are expressed as proportion of cells versus the infective dose (**A**) or versus 90 minute CFU (**B**). Student's *t*-test of wildtype versus mutant within each group was performed (** = p<0.05, *** = p<0.001) using GraphPad Prism 8.1.2. Error bars represent standard deviation from the mean of triplicate technical repeats. A representative figure from three independent biological replicates is shown.

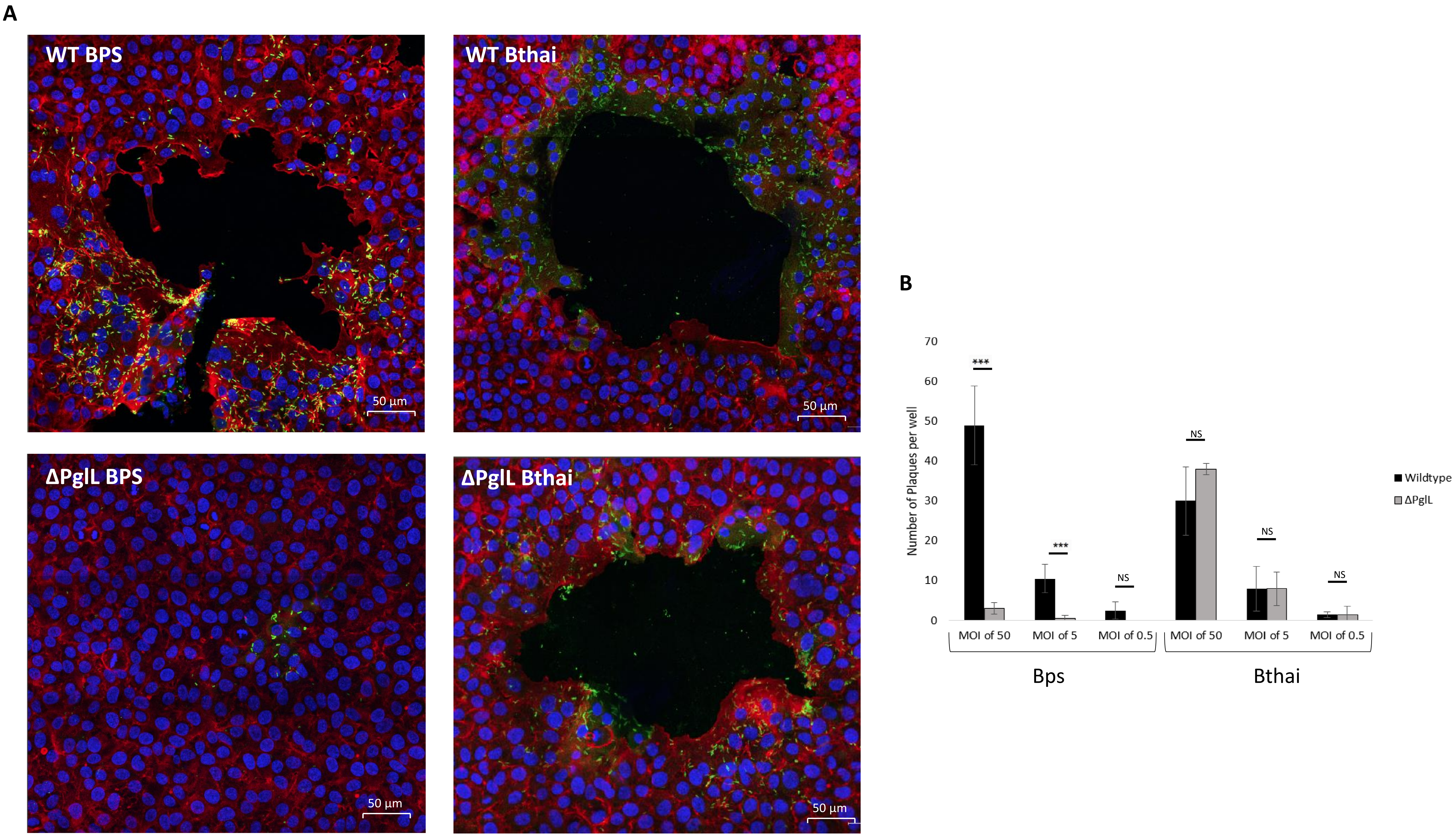

Figure 5: Plaque Formation by Wildtype and Δ*pglL Burkholderia spp.* In an A549 Cell Monolayer. *Burkholderia spp.* strains were used to infect a confluent monolayer of A549 cells in chamber slides. After 90 mins infection at different MOIs, cells were incubated in the presence of 100 ug mL^{-1} kanamycin for a further 16 hours. Immunofluorescence with confocal microscopy was used to image the monolayers, with nuclei stained with DAPI (blue), host-cell actin cytoskeleton was stained with phalloidin_alexafluor_546 (red) and bacteria visualised using an alexafluor_488 secondary antibody (**A**). For counting, plaques were visualised using phase-contrast microscopy and the entire well of duplicate wells were used for counting for each condition (**B**). Student's *t*-test of wildtype versus mutant was performed within each species and MOI group (*** = $p<0.001$) using GraphPad Prism 8.1.2. Error bars represent standard deviation from the mean of duplicate technical

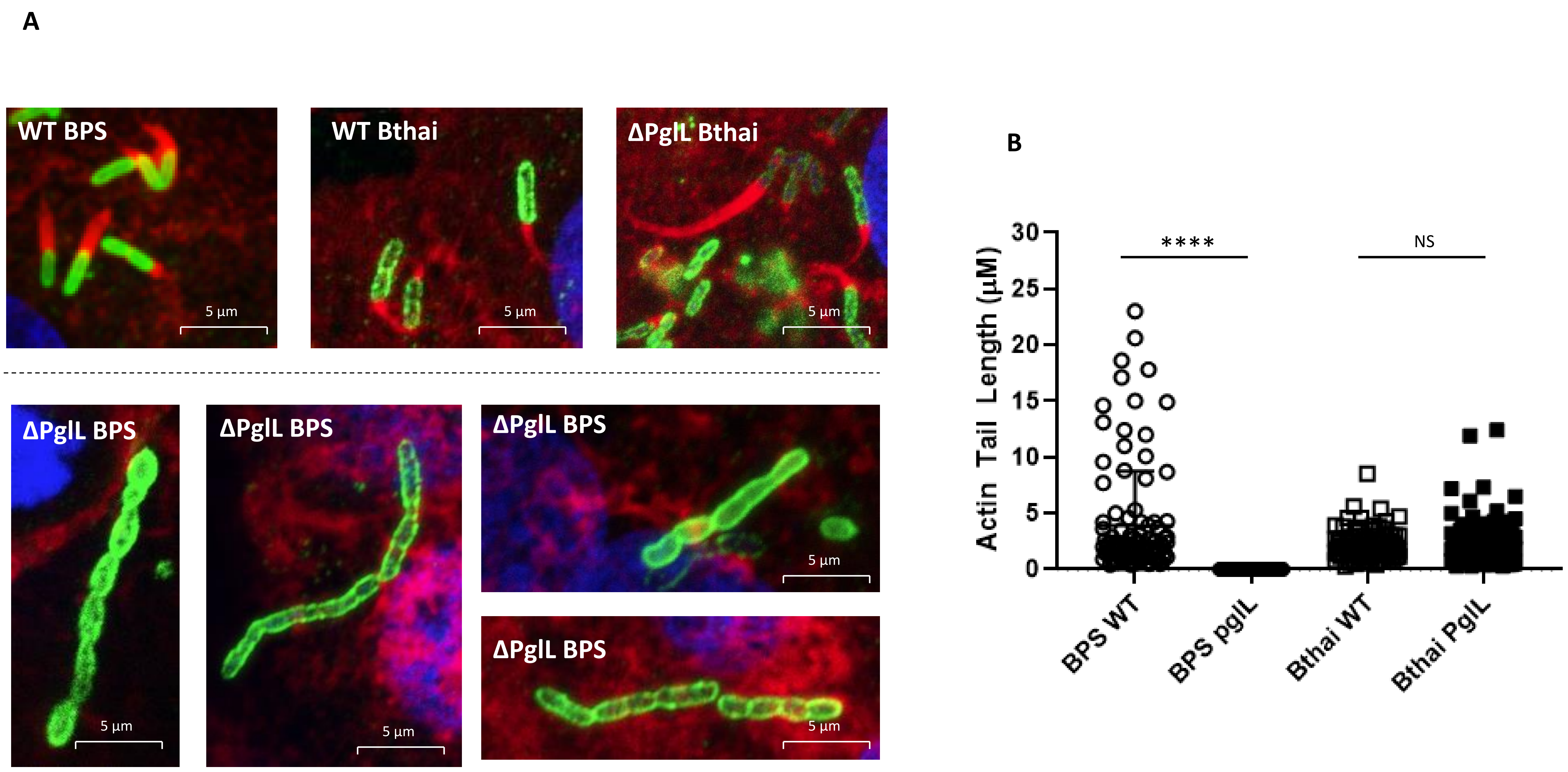

Figure 6: Actin-Tail Formation by Wildtype and Δ*pglL Burkholderia spp.* in an A549 Cell Monolayer. *Burkholderia spp.* strains were used to infect a confluent monolayer of A549 cells in chamber slides. After 90 mins infection at different MOIs, cells were incubated in the presence of 100 ug mL^{-1} kanamycin for a further 16 hours. Immunofluorescence with confocal microscopy was used to image the monolayers, with nuclei stained with DAPI (blue), host-cell actin cytoskeleton was stained with phalloidin_alexafluor_546 (red) and bacteria visualised using an alexafluor_488 secondary antibody (**A**). To measure actin tails, Zeiss Zen software was used, and the results displayed as a scatter chart (**B**). Mean lengths were: BPS WT 3916 nm (n=106); BPS Δ*pglL* 3 nm (n=43); Bthai WT 2204 nm (n=86); Bthai Δ*pglL* 2222 nm (n=112). Statistical difference between mutant and wildtype for each species was determined by Mann-Whitney test (****=$p<0.0001$) using GraphPad Prism 8.1.2.

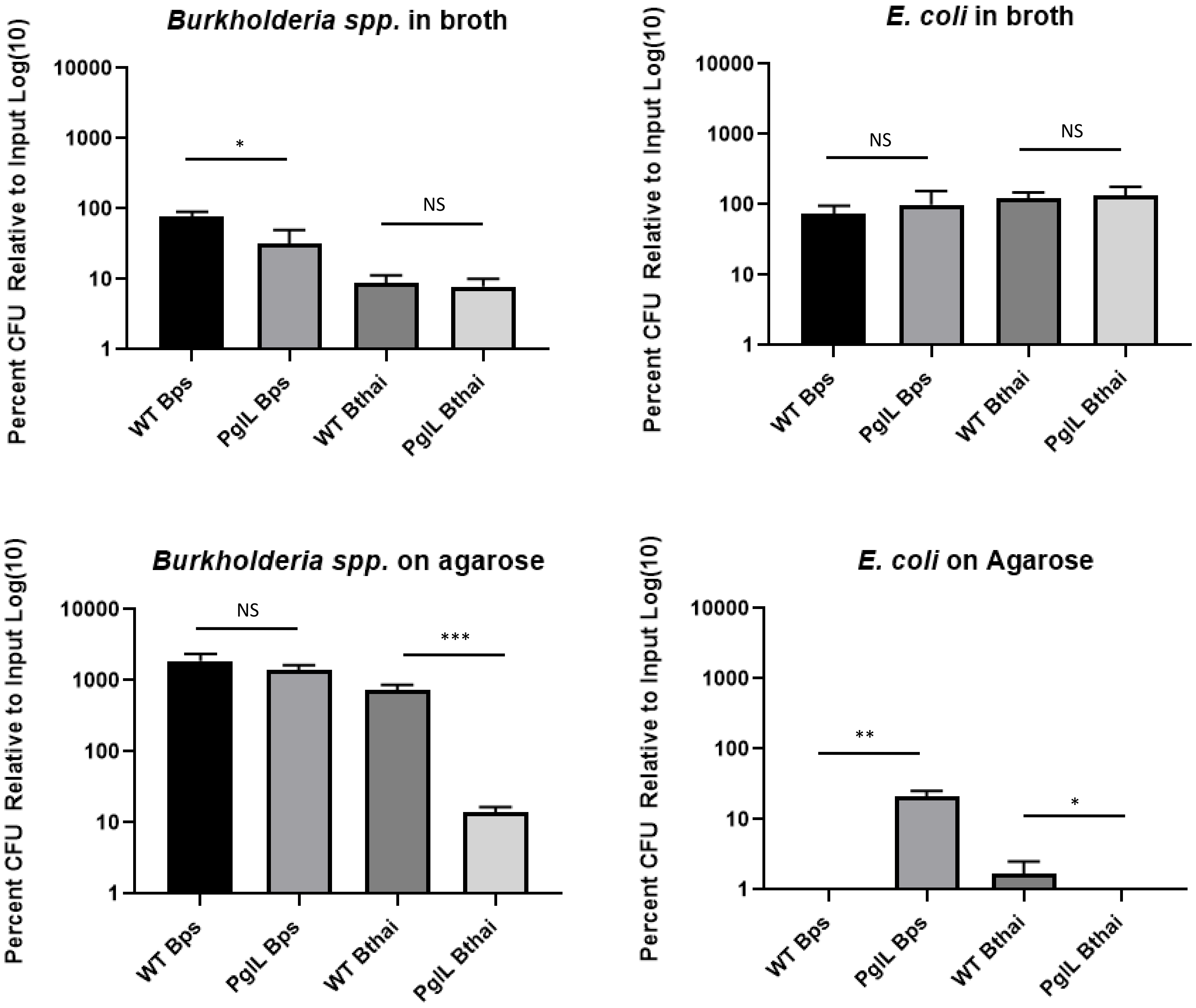

Figure 7: Competition Assay Between Wildtype or Δ*pglL Burkholderia spp.* and *E. coli*. *Burkholderia spp.* strains were co-cultured with *E. coli*_pcDNA 3.3 TOPO_*LacZ* at identical optical densities for five hours in either LB broth or spotted onto LB agar, as indicated. Bacteria were enumerated by CFU assay on agar plates coated with X-gal for distinguishing bacterial species by blue/white screening. Student's *t*-test was performed for each species' wildtype versus the mutant strain within each condition (*=p<0.05; ***p=<0.001) using GraphPad Prism 8.1.2. Error bars represent standard deviation from the mean of triplicate technical repeats. A representative figure from three independent biological replicates is shown.

A

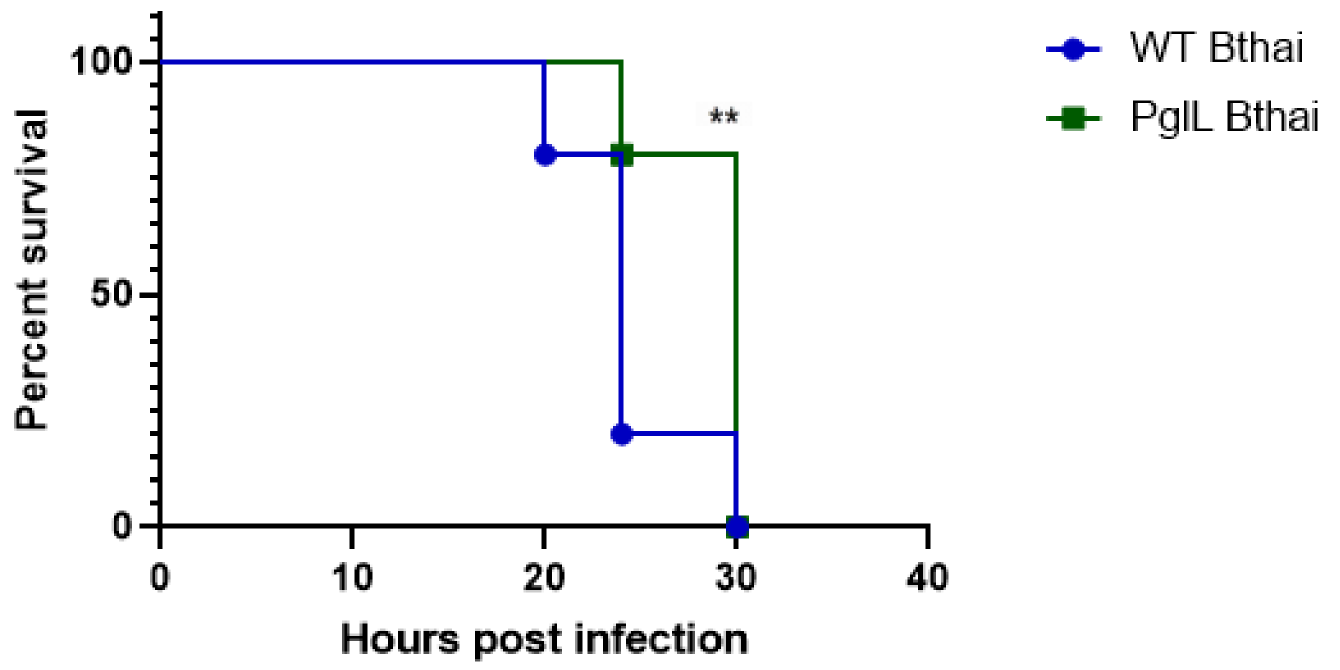

B

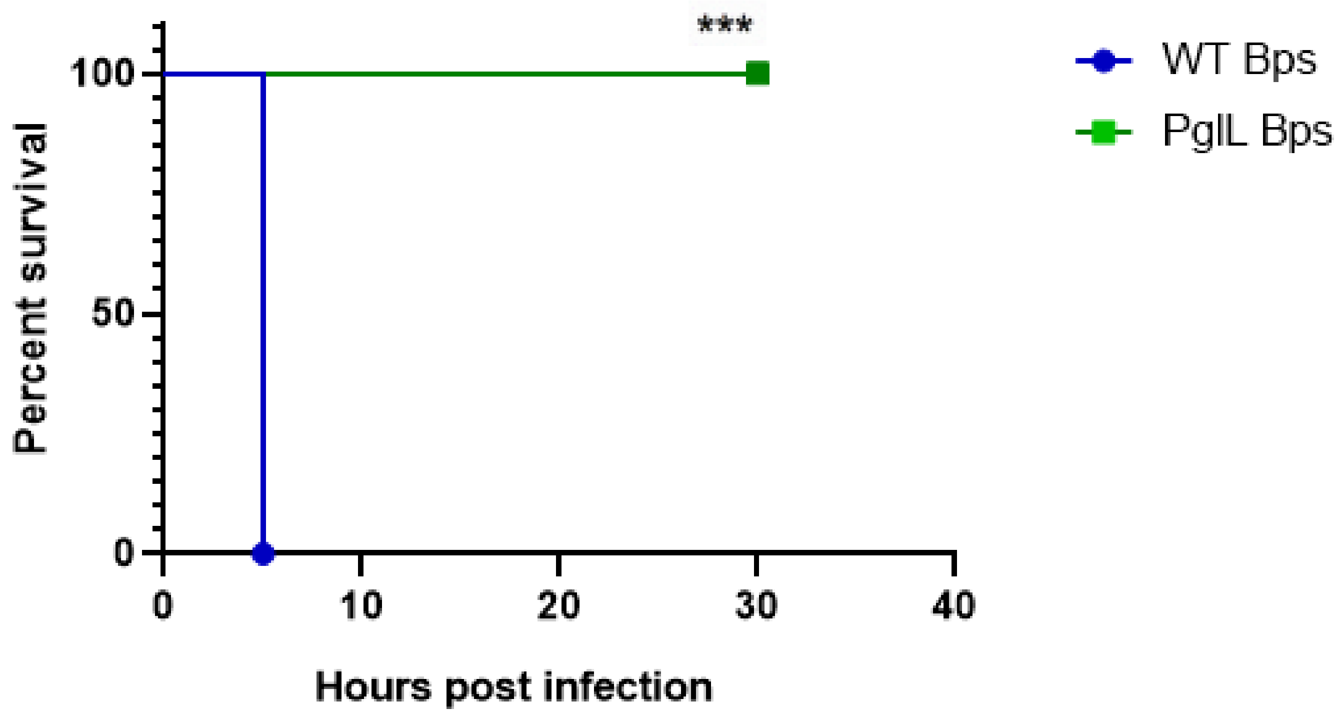

C

Organ	CFU	Organ	CFU
Lung 1	0	Spleen 1	0
Lung 2	0	Spleen 2	5904
Lung 3	0	Spleen 3	0
Lung 4	28	Spleen 4	122
Lung 5	0	Spleen 5	0

Figure 8: *In vivo* Virulence of Wildtype and Δ*pglL Burkholderia spp.* *G. mellonella* larvae (n=10 per group) were infected with 1,000 CFU Bthai wild-type (WT) or Bthai Δ*pglL* or inoculated with PBS alone. After 36 hours, difference between WT E264 survival was compared to mutant pglL (** P=< 0.05 Log-rank Mantel-Cox and Gehan-Breslow-Wilcoxon test) (**A**). Female BALB/C mice (n=5 per group) were infected intranasally with approximately 1000 CFU either wildtype BPS K92643 or BPS Δ*pglL* in sterile saline (*** p=<0.01 Log-rank Mantel-Cox and Gehan-Breslow-Wilcoxon test) (**B**). Surviving mice were culled at day 30 post-infection and total bacterial CFU in lungs and spleen enumerated (**C**).

A

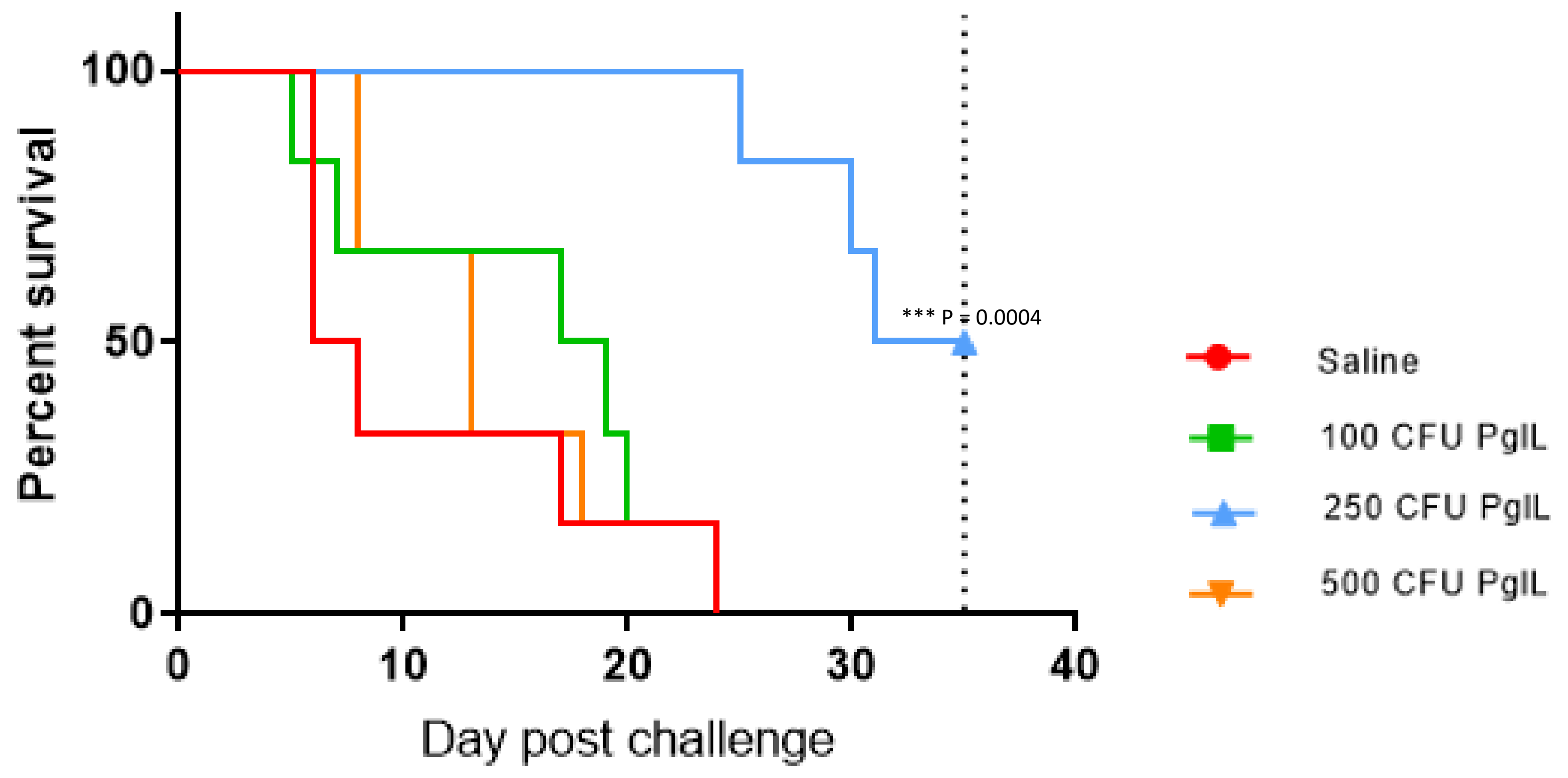

B

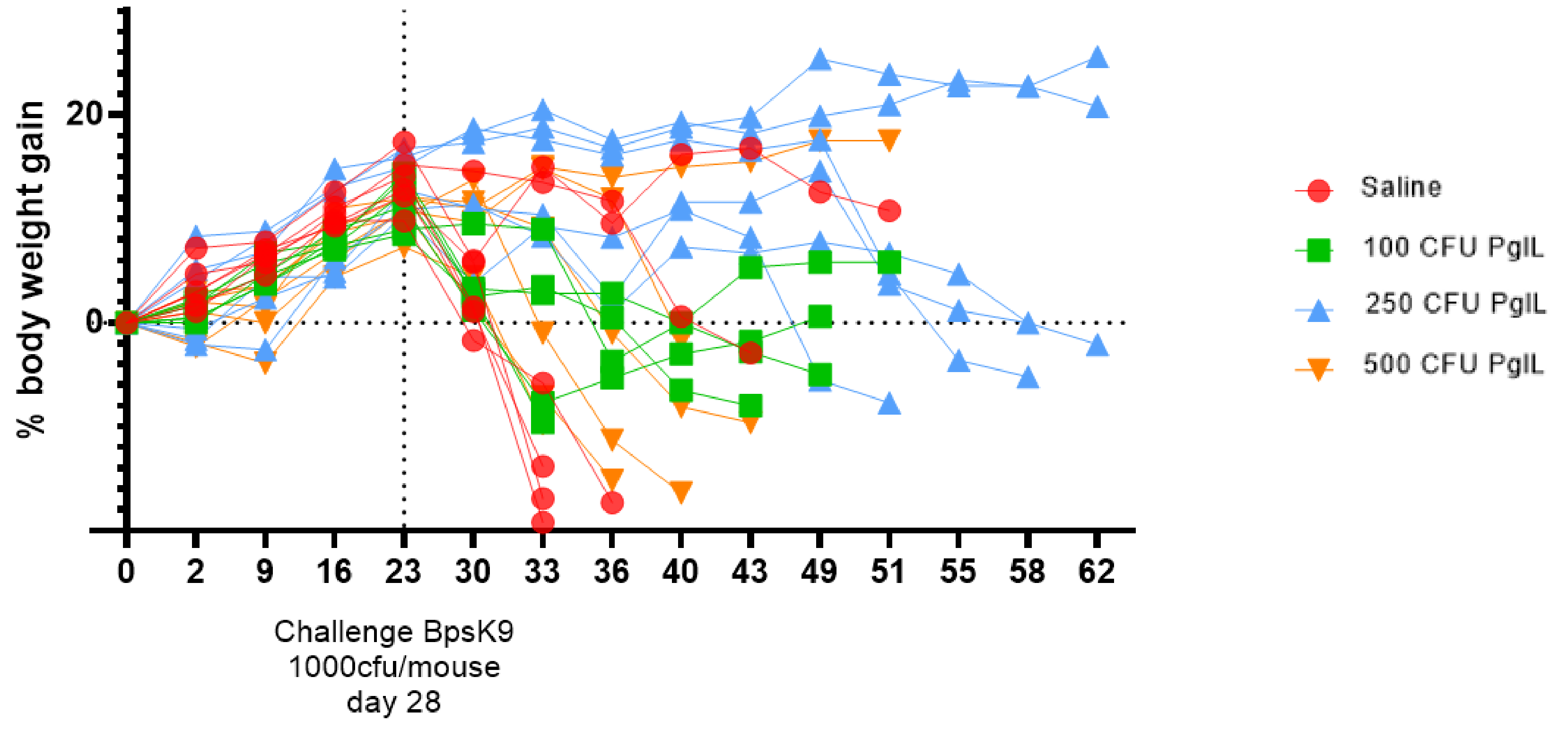

C

Organ	Wildtype:ΔPglL	Organ	Wildtype:ΔPglL
Lung 1	1:1	Spleen 1	1:1
Lung 2	1:1	Spleen 2	3:1
Lung 3	3:1	Spleen 3	ND

ND = excluded due to splenomegaly

Figure 9: Vaccination of Female BALB/C Mice with Δ*pglL B. pseudomallei* and Challenge with Wildtype BPS K9264. Female BALB/C mice (n=6 per group) were infected intranasally with different CFU of BPS Δ*pglL* as indicated, and after 28 days, challenged intranasally with wildtype BPS K9264 (*** p=<0.01 Log-rank Mantel-Cox and Gehan-Breslow-Wilcoxon test). Survival (**A**) and body weight (**B**) were monitored up to 68 days post-vaccination. At 68 days, surviving mice (from the 250 CFU vaccinated group) were culled and strains of organ-resident bacteria identified by polymerase chain reaction for the *pglL* gene to establish the ratio of wildtype (challenge strain) to Δ*pglL* (vaccine strain) colonies (**C**).

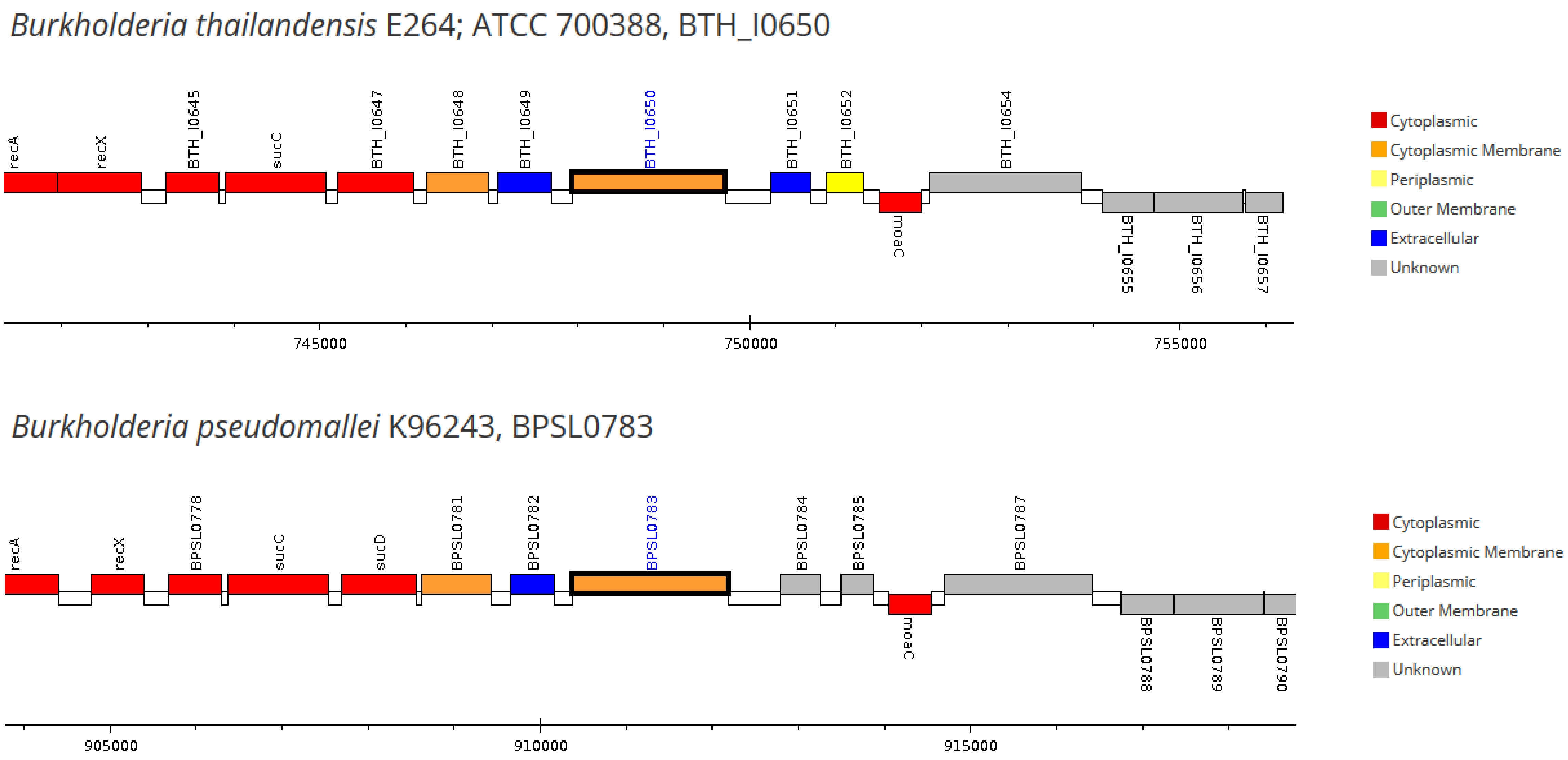

Figure S1: Genomic Context of *pglL* in *B. thailandensis* E264 and *B. pseudomallei* K9264. Genes were visualised using the www.burkholderia.com website.

Gene name	B.th ID	B.ps ID	Product annotation	Localisation
sucC	BTH_I0646	BPSL079	succinyl-CoA synthetase subunit beta	cytoplasmic
sucD	BTH_I0647	BPSL0780	succinyl-CoA synthetase subunit alpha	cytoplasmic
	BTH_I0648	BPSL0781	TerC family integral membrane protein	cytoplasmic membrane
pilA	BTH_I0649	BPSL0782	pilin family protein	extracellular
pglL	BTH_I0650	BPSL0783	O-antigen polymerase family protein	cytoplasmic membrane
	BTH_I0651	BPSL0784	hypothetical protein	extracellular
	BTH_I0652	BPSL0785	TonB domain-containing protein	periplasmic
moaC	BTH_I0653	BPSL0786	molybdenum cofactor biosynthesis protein MoaC	cytoplasmic
	BTH_I0654	BPSL0787	M48 family peptidase	unknown

Table S1: Identity of Upstream and Downstream Flanking Genes of *pglL* in *B. thailandensis* E264 and *B. pseudomallei* K9264.

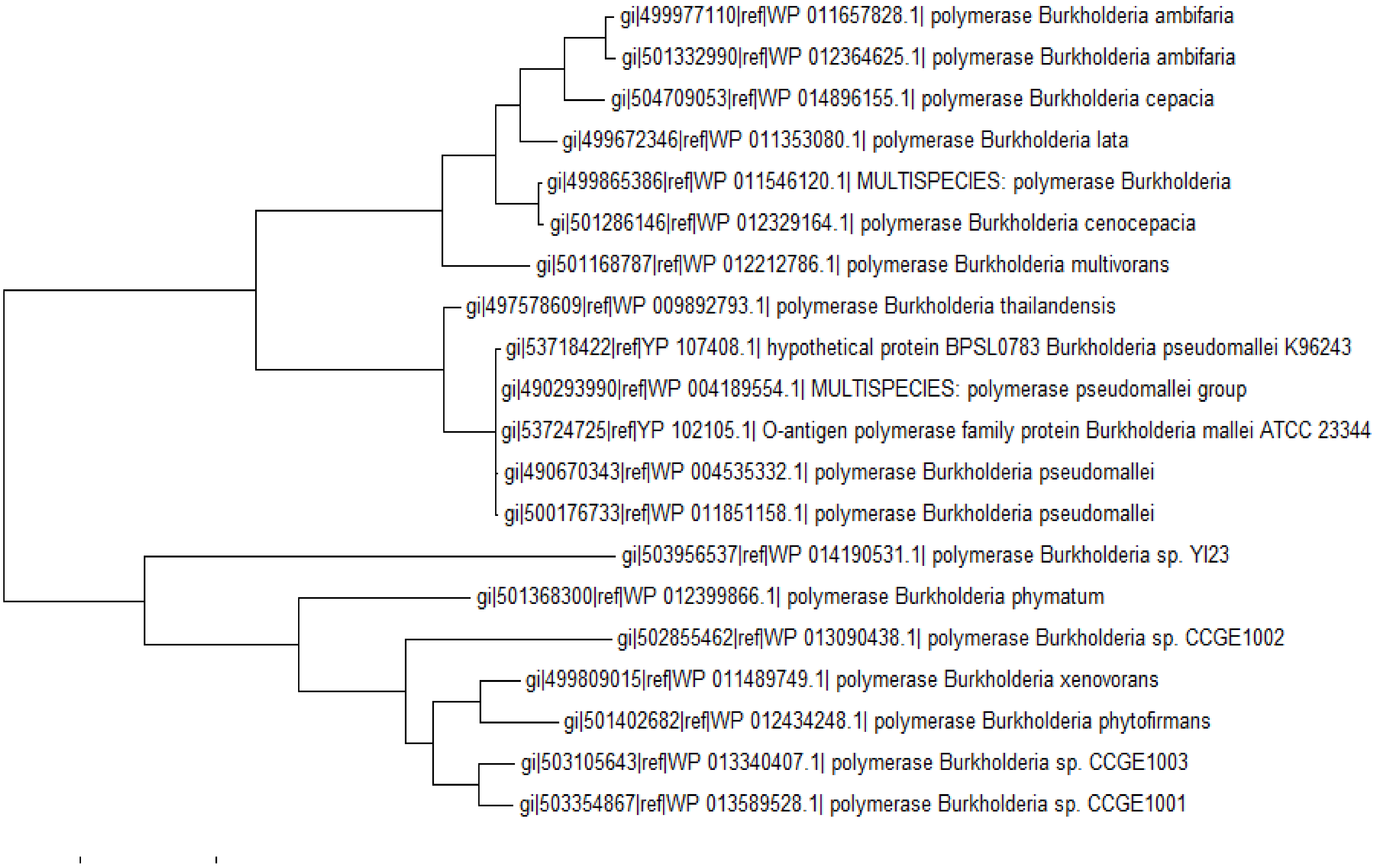

Figure S2: Phylogenetic Analysis of *pglL*. Sequences were obtained from the www.burkholderia.com database and tree assembled using MEGA4 software.

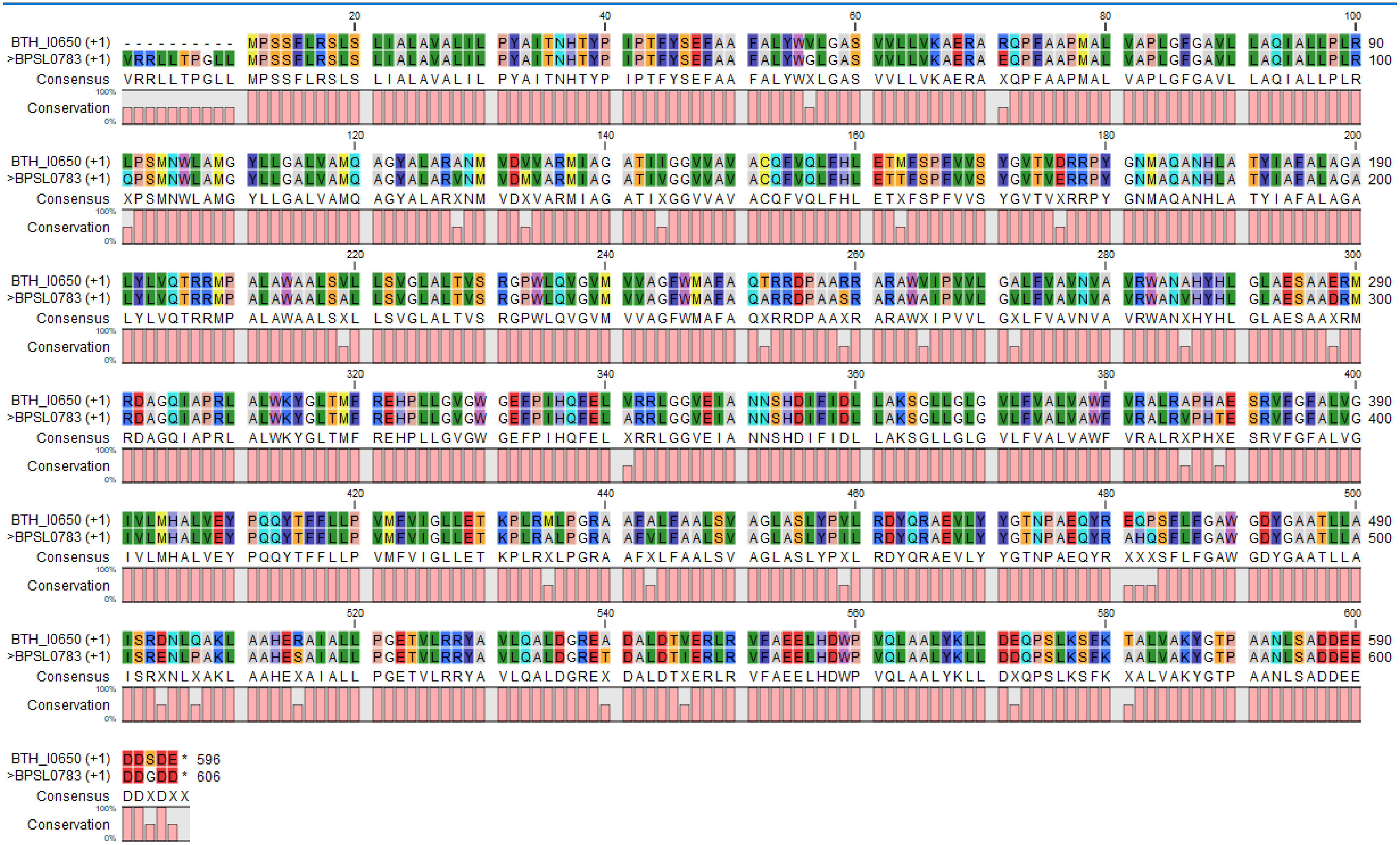

Figure S3: Amino-Acid Homology Comparison Between *B. Thailandensis* and *B. pseudomallei* pglL. Alignments were made using CLC Sequence Viewer.

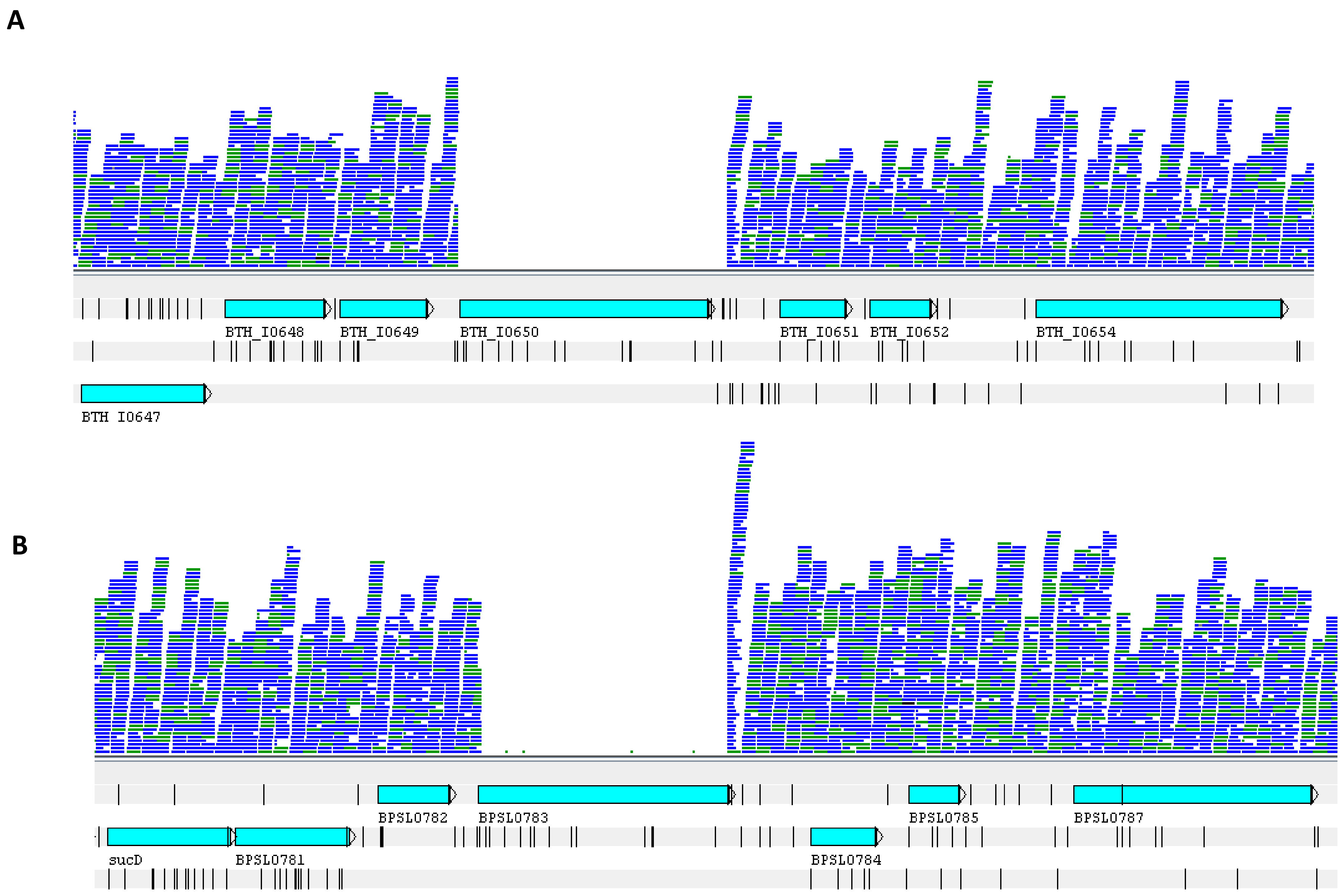

Figure S4: Whole-genome Next Generation Sequencing of *Burkholderia spp.* Δ*pglL* mutants. Independent colonies representing Δ*pglL* deletion mutants from each species were selected for genomic DNA extraction and next generation sequencing (performed by Public Health England, Genomic Services and Development Unit, UK). Raw sequence data was mapped to the reference genomes for each species: *B. thailandensis* E264 CP000086 (**A**) and *B. pseudomallei* K9264 BX571965 (**B**) using Artemis Release 16.0.0 software. The specific region for the *pglL* gene is shown in a representative image from each species, demonstrating a clean, marker-less deletion at the site of interest.
